# Parental conflict driven regulation of endosperm cellularization by a family of Auxin Response Factors

**DOI:** 10.1038/s41477-024-01706-y

**Published:** 2024-05-28

**Authors:** N. Butel, Y. Qiu, W. Xu, J. Santos-González, C. Köhler

**Affiliations:** 1 Department of Plant Reproductive Biology and Epigenetics, Max Planck Institute of Molecular Plant Physiology, Potsdam, Germany; 2 Department of Plant Biology, Uppsala BioCenter, Swedish University of Agricultural Sciences and Linnean Centre for Plant Biology, Uppsala, Sweden; 3 Present Address: INRAE Centre Ile-de-France - Versailles-Saclay, France, Versailles-Sacley, France

**Keywords:** Biological techniques, Cell biology

## Abstract

The endosperm is a reproductive tissue supporting embryo development. In most flowering plants, the initial divisions of endosperm nuclei are not succeeded by cellularization; this process occurs only after a specific number of mitotic cycles have taken place. The timing of cellularization significantly influences seed viability and size. Previous research implicated auxin as a key factor in initiating nuclear divisions and determining the timing of cellularization. Here we uncover the involvement of a family of clustered auxin response factors (cARFs) as dosage-sensitive regulators of endosperm cellularization. *cARFs*, maternally expressed and paternally silenced, are shown to induce cellularization, thereby restricting seed growth. Our findings align with the predictions of the parental conflict theory, suggesting that *cARFs* represent major molecular targets in this conflict. We further demonstrate a recurring amplification of *cARFs* in the Brassicaceae, suggesting an evolutionary response to parental conflict by reinforcing maternal control over endosperm cellularization. Our study highlights that antagonistic parental control on endosperm cellularization converges on auxin biosynthesis and signalling.

## Abstract (web summary)

In most flowering plants, early divisions of endosperm nuclei are not succeeded by cellularization. This study uncovered a family of clustered auxin response factors as dosage-sensitive, maternally expressed regulators of endosperm cellularization.

## Main

The endosperm is a reproductive tissue derived from the fusion of a haploid sperm cell with a predominantly diploid central cell, which sustains and supports embryo development^1^.

In *Arabidopsis thaliana*, as in most angiosperms, endosperm development occurs in two phases. In the initial phase, endosperm nuclei proliferation is not followed by cellularization, resulting in the formation of a coenocyte^2^. At a tightly controlled timepoint, a wave of cellularization starts from the micropylar region surrounding the embryo to reach the opposite chalazal endosperm^2^. At the end of the process, most of the endosperm is cellularized and nuclear divisions cease. The timing of the transition from the first to the second phase is critical for seed development. Precocious or delayed cellularization leads to very small or enlarged seeds of impaired viability, respectively^3^. Endosperm cellularization is under differential parental control; while increased maternal genome dosage promotes cellularization, increased paternal genome dosage has the opposite effect by delaying cellularization.

Previous work identified auxin as a critical factor initiating the first nuclear divisions of the endosperm and determining the timing of endosperm cellularization^4,5^. Auxin biosynthesis is initiated after fertilization from the paternal genome by *YUCCA10* (also known as *YUC10*) and *TRYPTOPHAN AMINOTRANSFERASE RELATED 1* (also known as *TAR1*), two imprinted paternally expressed genes regulating auxin biosynthesis^4^. Auxin levels cease at the time of cellularization, while conversely, endosperm cellularization failure correlates with increased auxin levels^4^. How auxin controls endosperm cellularization is nevertheless unknown.

## Results

We previously identified a cluster of Auxin Response Factors (ARFs) that is strongly upregulated in seeds with delayed endosperm cellularization^4,5^. Given the connection between auxin and endosperm cellularization, we investigated the function of those ARFs in the endosperm.

**Fig. 1:**
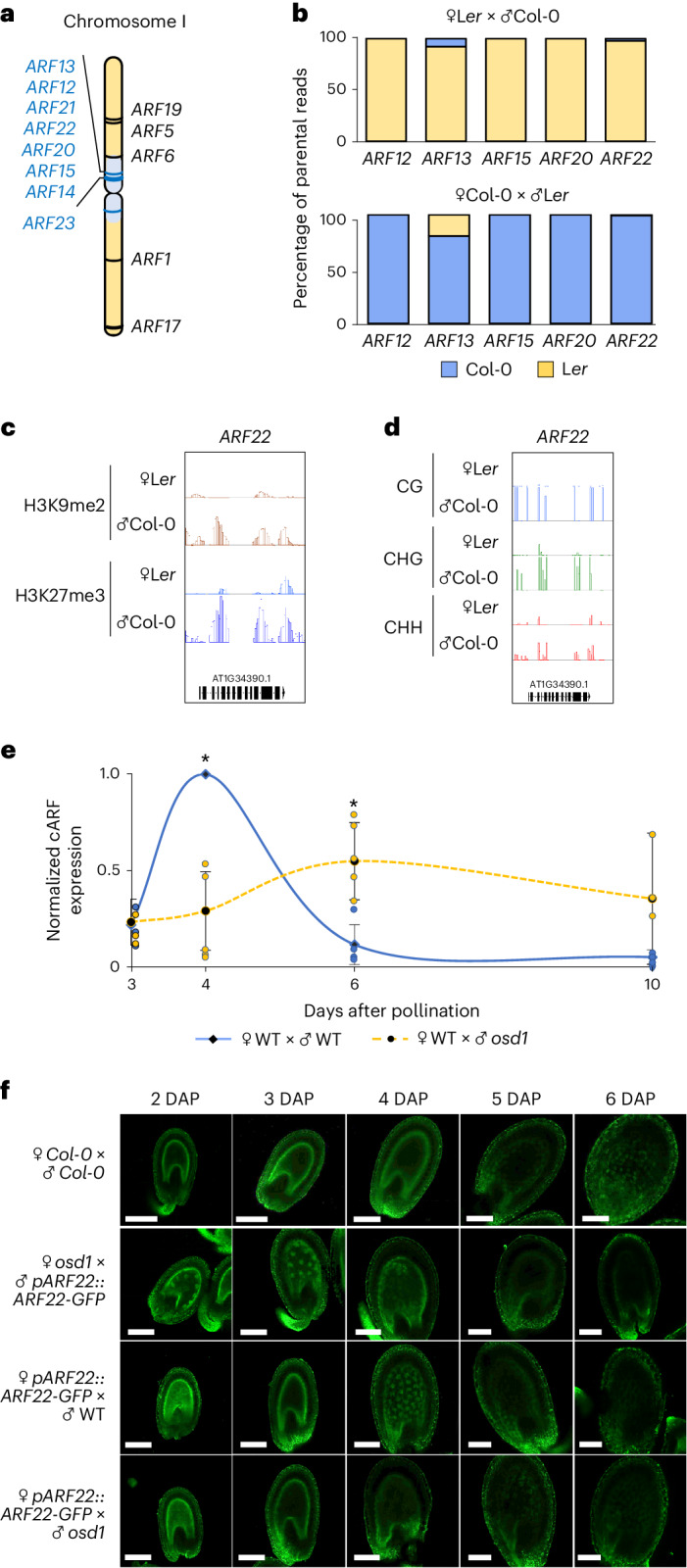
*cARFs* are expressed at the onset of endosperm cellularization. **a**, Localization of *Arabidopsis* *ARF* genes on chromosome 1. Pericentromeric regions are highlighted in blue^37^, and *cARFs* are indicated with a blue line. **b**, Percentage of parental *cARF* reads derived from crosses of Col-0 and Landsberg *erecta* (L*er*) accessions in the 4 DAP endosperm^7^. **c**, Parental-specific enrichment of H3K9me2 (red) and H3K27me3 (blue) histone marks on *ARF22* in the 4 DAP endosperm^34^. **d**, Parental-specific DNA methylation in CG, CHG and CHH context (H stands for any base except G) on *ARF22* in the endosperm at 6 DAP^35^. **e**, RT–qPCR analysis of *cARF* expression in 3, 4, 6 and 10 DAP siliques of the indicated crosses. Data show mean ± s.d. of 5 independent biological replicates. *P_4DAP_ = 0.003772; *P_6DAP_ = 0.01584 (two-sided Student’s *t*-test). **f**, Confocal microscopy pictures showing expression of *pARF22:ARF22-GFP* at different stages of seed development in the indicated crosses. Data are based on 2 biological replicates with a minimum of 30 seeds per replicate. Scale bars, 100 µm.

**Extended Data Fig. 1:**
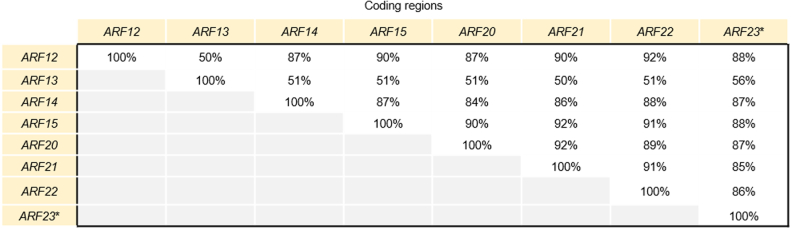
cARFs share high sequence similarity. Percentage of identity at the protein level between each cARF. *ARF23* is a pseudogene, indicated by the asterisk. The analysis was done using Clustal Omega.

This *ARF* cluster contains eight members that are located in the pericentromeric region of chromosome 1 (Fig. 1a). All members share high sequence similarity, indicating that they function redundantly (Extended Data Fig. 1 and Supplementary Information). The exceptions are *ARF13* for which the sequence has diverged, and *ARF23* which is truncated and has been proposed to be a pseudogene and was therefore not considered further^6^. We will refer to these clustered *ARFs* as *cARFs*.

**Extended Data Fig. 2:**
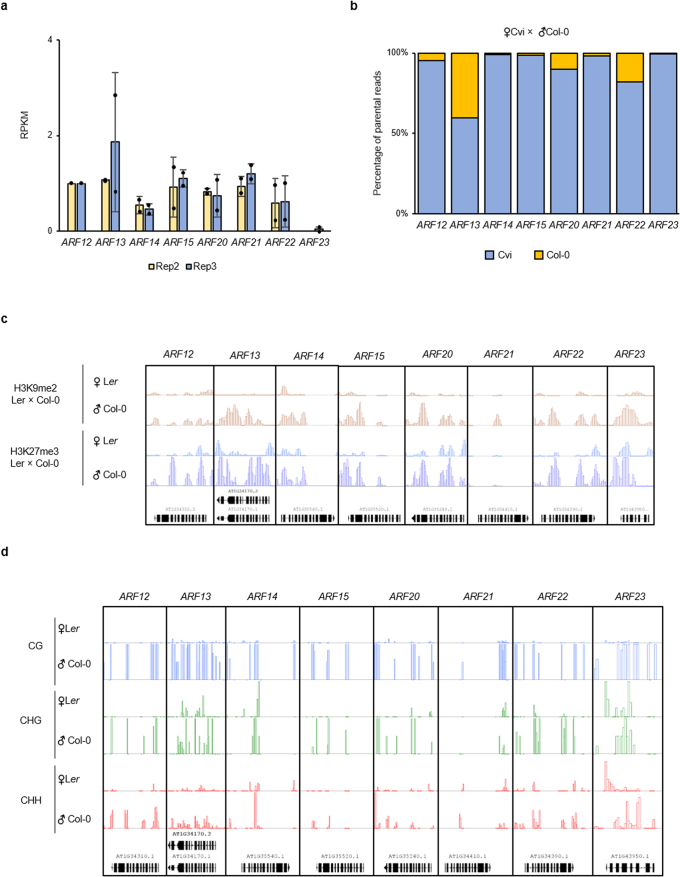
The paternal allele of *cARFs* is marked by repressive histone modifications and DNA methylation. (**a**) Individual *cARF* expression in the endosperm at 4DAP^7^. Data show the mean expression values of two independent crosses, with error bars representing the standard deviation. (**b**) Percentage of parental *cARF* reads derived from crosses of Cvi and Col-0 accessions in the 4 DAP endosperm^36^. (**c**) Parental-specific enrichment of H3K9me2 (red) and H3K27me3 (blue) histone modifications on *cARFs* in the 4 DAP endosperm^34^. (**d**) Bedgraphs showing parental-specific DNA methylation in the endosperm at 6DAP^35^.

Based on available transcriptome data of the endosperm 4 days after pollination (DAP)^7^, *cARFs* are expressed at similar levels, suggesting that they are functionally redundant (Extended Data Fig. 2a). Based on available parental-specific endosperm transcriptome data, all *cARFs* are maternally expressed genes (MEGs), thus the maternal alleles are exclusively or preferentially expressed in the endosperm (Fig. 1b and Extended Data Fig. 2b). The paternal alleles of all *cARFs* are highly DNA methylated and enriched for repressive histone methylation on H3 lysine 27 and lysine 9 (H3K27me3 and H3K9me2, respectively), correlating with the specific silencing of the paternal alleles (Fig. 1c,d and Extended Data Fig. 2c,d).

### *cARFs* are expressed at the onset of endosperm cellularization

**Extended Data Fig. 3:**
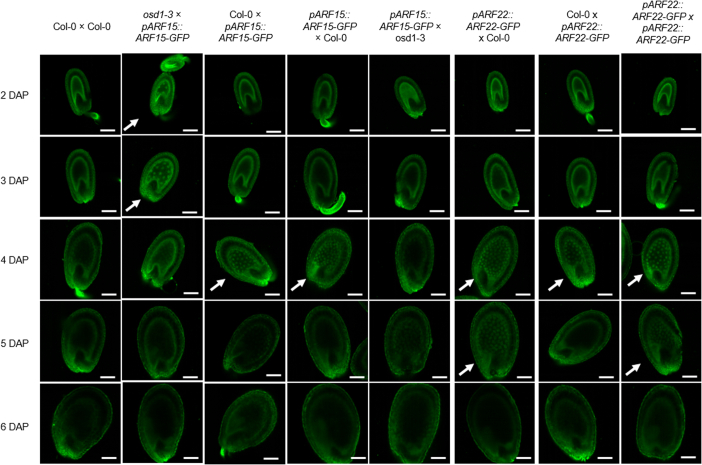
*pARF15::ARF15-GFP* and *pARF22::ARF22-GFP* show the same expression pattern. (**a**) Confocal microscopy pictures showing expression of *pARF15::ARF15-GFP* and *pARF22::ARF22-GFP* at different stages of seed development in the indicated crosses. Data are based on two biological replicates and 30 seeds were analyzed per replicate. Scale bars, 100 µm.

Previous reports found *cARFs* to be expressed in the micropylar endosperm domain at the globular stage of embryo development^8–10^. To specifically determine when and where *cARFs* are expressed, we monitored transcript abundance by quantitative PCR with reverse transcription (RT–qPCR) and protein localization using reporter constructs for *ARF15* and *ARF22*, which contain the promoter and coding region of both genes fused to the green fluorescent protein (GFP) reporter (*pARF15::ARF15-GFP* and *pARF22::ARF22-GFP*) (Fig. 1e,f and Extended Data Fig. 3). Since *cARFs* are highly similar at nucleotide sequence level (Extended Data Fig. 1 and Supplementary Information), discriminating individual *cARFs* by RT–qPCR was not possible. We thus monitored transcript levels of all *cARFs* and found them to peak at 4 DAP (Fig. 1e). Similarly, GFP fluorescence accumulated in both the micropylar and the peripheral endosperm at ~4–5 DAP (Fig. 1e,f and Extended Data Fig. 3). Thus, cARF accumulation preceded endosperm cellularization, which in *Arabidopsis* wild-type Col-0 initiated at 5–6 DAP.

In seeds inheriting a double dosage of paternal chromosomes (referred to as paternal excess crosses), *cARFs* were deregulated^5^, suggesting that *cARFs* are sensitive to parental genome dosage. To test this hypothesis, we monitored *pARF15::ARF15-GFP* and *pARF22::ARF22-GFP* expression in seeds with unbalanced parental genome dosage. We made use of the *omission of second division 1 (osd1)* mutant that produces 2n male and female gametes at high frequency^11^. Thus, using *osd1* as either the female or the male parent allowed generation of seeds with either increased maternal or paternal genome dosage, correlating with precocious (4–5 DAP) or delayed endosperm cellularization (after 6 DAP), respectively^3^.

We found that increased paternal genome dosage generated by crossing wild-type (WT) plants with *osd1* pollen donors caused reduced and delayed *cARF* transcript accumulation, shifting the peak of expression from 4 to 6 DAP (Fig. 1e). This pattern was also reflected by the *pARF22::ARF22-GFP* and the *pARF15::ARF15-GFP* reporters; we did not detect GFP fluorescence in paternal excess seeds between 2 and 6 DAP (Fig. 1f and Extended Data Fig. 3).

Conversely, in maternal excess seeds where *osd1* was the female parent, *ARF22-GFP* and *ARF15-GFP* expression could be already detected at 2–3 DAP (Fig. 1f and Extended Data Fig. 3). This early expression was not a consequence of increased copy number, since the constructs are not imprinted and introduced through pollen. We failed to detect *cARF* transcripts in maternal excess seeds by RT–qPCR, probably because the endosperm nuclei number was too low to allow detection of low-abundance endosperm transcripts. Nonetheless, the detection of precocious *ARF22-GFP* and *ARF15-GFP* activity strongly suggests that *cARF* expression is sensitive to maternal genome dosage and that increased maternal genome dosage correlates with increased *cARF* expression.

Together, these results show that *cARF* expression is antagonistically regulated by maternal and paternal genome dosage, reflecting their MEG identity. Furthermore, *cARF* activity correlates with the onset of endosperm cellularization^3^, suggesting a functional role of cARFs in regulating this process.

### cARF deficiency delays endosperm cellularization

**Fig. 2:**
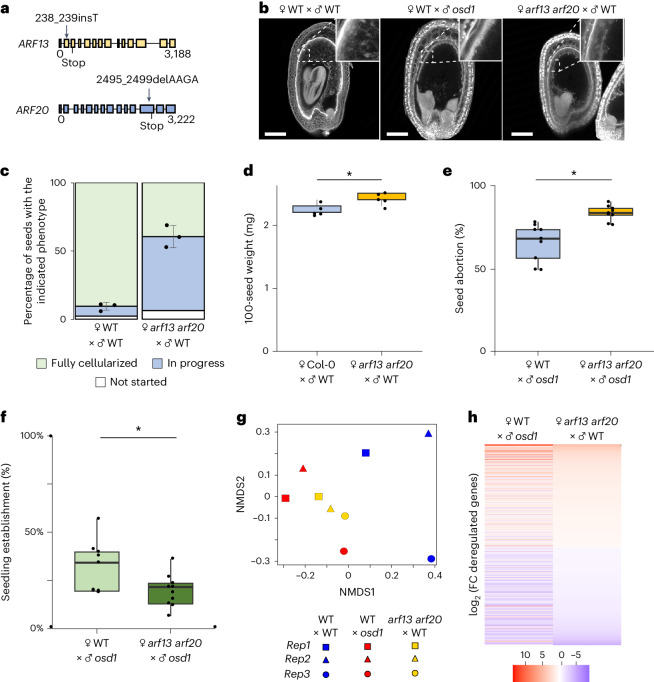
Mutations in *cARFs* delay endosperm cellularization. **a**, Schematic representation of *ARF13* and *ARF20* and positions of two mutations induced by CRISPR/Cas9. The filled squares correspond to exons. **b**, Multiphoton microscopy pictures of 7 DAP Feulgen-stained seeds derived from indicated crosses. Scale bars, 100 µm. **c**, Quantification of endosperm cellularization in seeds of indicated crosses. ‘In progress’ refers to seeds where cellularization has initiated but not terminated (see Extended Data Fig. 8 for details). Data show mean ± s.d. of 3 independent biological replicates, with a minimum of 50 seeds per replicate. **d**, The 100-seed weight of seeds derived from the indicated crosses. Each dot represents the weight of 100 seeds. Five independent measurements were analysed for each line. *P = 0.019 (two-sided Student’s *t*-test). **e**, Percentage of aborted seeds derived from indicated crosses. **f**, Percentage of established seedlings from seeds of the indicated crosses. **e**,**f**, Each dot represents the percentage of aborted seeds (**e**) or established seedlings (**f**) from 3–5 siliques. Data are based on 3 biological replicates, each comprising 3 inflorescences, resulting in a total of 9 values. *P_seed abortion_ = 0.000468; *P_seedling establishment_ = 0.0409 (two-sided Student’s *t*-test). **d**–**f**, Boxes show median values and the interquartile range. Whiskers show minimum and maximum values, excluding outliers. **g**, NMDS multivariate analysis of transcriptomes of 7 DAP seeds of the indicated genotypes. **h,** Heat map showing the log_2_ (fold change) (FC) of deregulated genes in *arf13 arf20* × WT compared to WT, and WT × *osd1* compared to WT at 7 DAP. Only genes that were significantly deregulated in WT × *osd1* compared with WT after multiple-testing correction (|log_2_ FC| ≥ 1; *P*_adj_ < 0.05) are shown.

**Extended Data Fig. 4:**
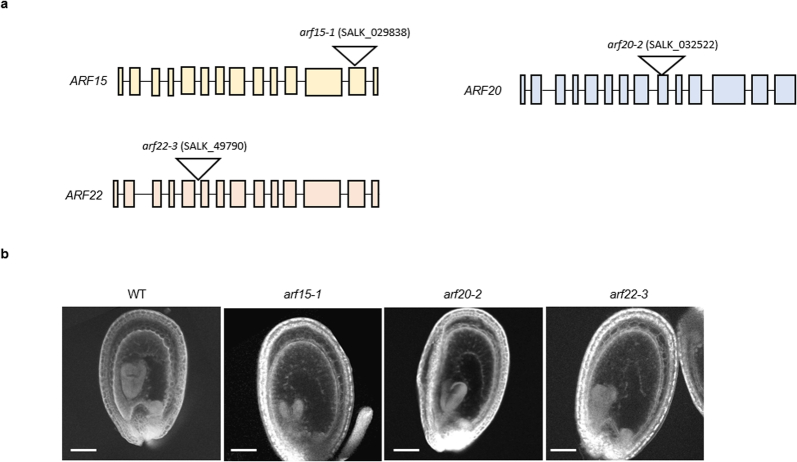
Single *arf* T-DNA insertion mutants do not exhibit abnormal seed phenotypes. (**a**) Schematic representation showing the position of T-DNA insertions in *ARF15*, *ARF20* and *ARF22*. Filled boxes correspond to exons. (**b**) Multiphoton microscopy pictures of 6 DAP Feulgen stained seeds. A minimum of 30 seeds were analyzed. Scale bars, 100 µm.

**Extended Data Fig. 5:**
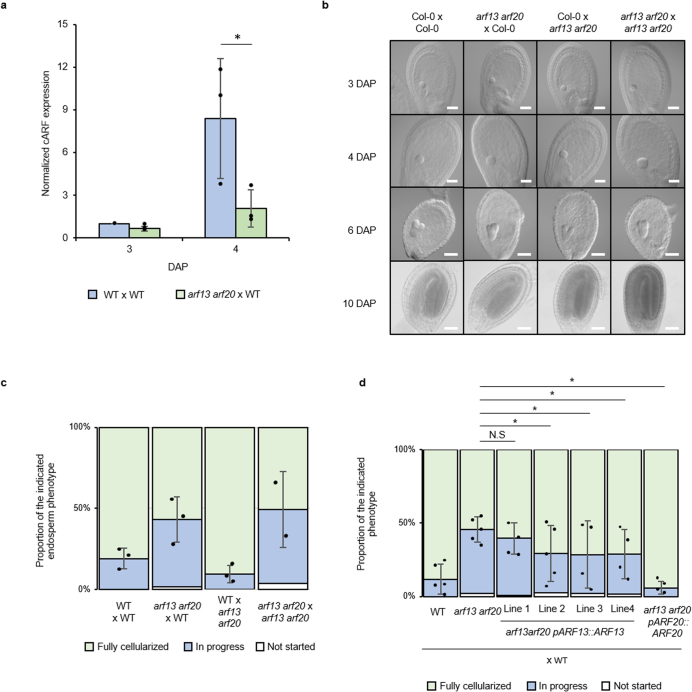
Characterization of the *arf13 arf20* line. (**a**) RT-qPCR analysis of *cARF* expression in 3- and 4-DAP siliques of the indicated crosses. Data show the mean of three independent biological replicates (n = 3), with error bars indicating the standard deviation. Asterisks denote statistically significant differences based on a one-sided Student’s t-test. (*P = 0.03415). (**b**) Pictures of cleared seeds taken at the indicated time points. Data are based on two biological replicates and 30 seeds were analyzed per replicate. Scale bars, 100 µm. (**c**) Quantification of endosperm cellularization in seeds of indicated crosses. In progress refers to seeds where cellularization has initiated but not terminated (See Extended Data Figure 8 for pictures). Data show the mean of three independent biological replicates (n = 3) and a minimum of 50 seeds were analyzed per replicate. Error bars represent standard deviation. (**d**) Quantification of endosperm cellularization in seeds of indicated crosses. Data show the mean of five independent biological replicates (n = 5) and a minimum of 50 seeds were analyzed per replicate. Error bars represent standard deviation. Asterisks denote statistically significant differences based on a Chi-squared test with Bonferroni correction. (*P_ARF13-2_ = 7.08 × 10-5; *P_ARF13-3_ = 8.675 × 10-5; *P_ARF13-4_ = 2.314 × 10-4; *P_ARF20_ = 0).

Single T-DNA insertions in *ARF15, ARF20* and *ARF22* did not cause abnormalities in seed development, suggesting functional redundancy of cARFs (Extended Data Fig. 4). Using CRISPR/Cas9 with two guide RNAs targeting multiple *cARFs*, we identified one line with premature stop codons in *ARF13* and *ARF20*, reflected by reduced *cARF* transcript levels at 4 DAP (Fig. 2a and Extended Data Fig. 5a).

Since *ARF13* and *ARF20* are predominantly maternally expressed, we pollinated *arf13 arf20* with WT pollen to test the effect on endosperm cellularization. Loss of maternal ARF13 ARF20 function did not affect embryo development (Extended Data Fig. 5b) but delayed endosperm cellularization; while most wild-type seeds were completely cellularized at 7 DAP, the majority of *arf13/+ arf20/+* seeds had only started the cellularization process, resembling paternal excess seeds (Fig. 2b,c). Assessing the extent of endosperm cellularization poses challenges due to its occurrence in a three-dimensional context, rendering a single image insufficient for quantitative analysis. To quantitatively assess the degree of endosperm cellularization, we categorized seeds on the basis of the progression of the cellularization status as either not started, in progress, or fully cellularized. Using confocal imaging, we analysed multiple layers of Feulgen-stained seeds that formed the basis for this assessment. Delayed cellularization was not observed when *arf13 arf20* was paternally inherited, consistent with *cARFs* being MEGs (Extended Data Fig. 5c). The timing of the cellularization was completely or partially normalized when the mutants were complemented with a *pARF20::ARF20* or a *pARF13::ARF13* construct, respectively, confirming that mutations in *ARF13* and *ARF20* are responsible for the delayed cellularization phenotype (Extended Data Fig. 5d). Consistent with the delay of endosperm cellularization, seeds of *arf13 arf20* × Col-0 crosses were significantly heavier than the corresponding WT seeds (Fig. 2d). Together, these results reveal that maternal *cARFs* have a functional role in endosperm cellularization and probably induce cellularization.

In paternal excess seeds, *cARF* expression was delayed and reduced (Fig. 1e,f). To test the causality between *cARF* expression and the paternal excess phenotype, we tested whether the *arf13 arf20* mutant enhances the paternal excess phenotype. Indeed, the triploid seed abortion rate was higher when the *arf13 arf20* was used as the maternal parent compared with WT plants, corresponding to a reduced number of viable triploid *arf13 arf20* seedlings (Fig. 2e,f). Thus, impairing *cARF* function aggravates the paternal excess seed phenotype, consistent with a proposed role of cARFs in regulating endosperm cellularization.

To test whether the delay of endosperm cellularization in *arf13 arf20* and paternal excess seeds has a common molecular basis, we compared the transcriptomes of seeds lacking maternal ARF13 ARF20 function with paternal excess seeds at 7 DAP, when the corresponding wild type was fully cellularized. Indeed, we found that the transcriptomes of paternal excess seeds and *arf13 arf20* seeds clustered together, whereas the wild-type transcriptomes clustered separately (Fig. 2g and Supplementary Data 1). The similarity in transcriptomes was also reflected by a similar trend of deregulated genes in paternal excess seeds and seeds lacking ARF13 ARF20 function (|log_2_ FC| ≥ 1; *P*_adj_ < 0.05) (Fig. 2h).

Together, the transcriptional response in seeds lacking ARF13 and ARF20 function resembled that of paternal excess seeds, supporting the hypothesis that delayed cellularization in paternal excess seeds is linked to the misregulation of *cARFs*.

### *cARF* overexpression induces early cellularization

**Fig. 3:**
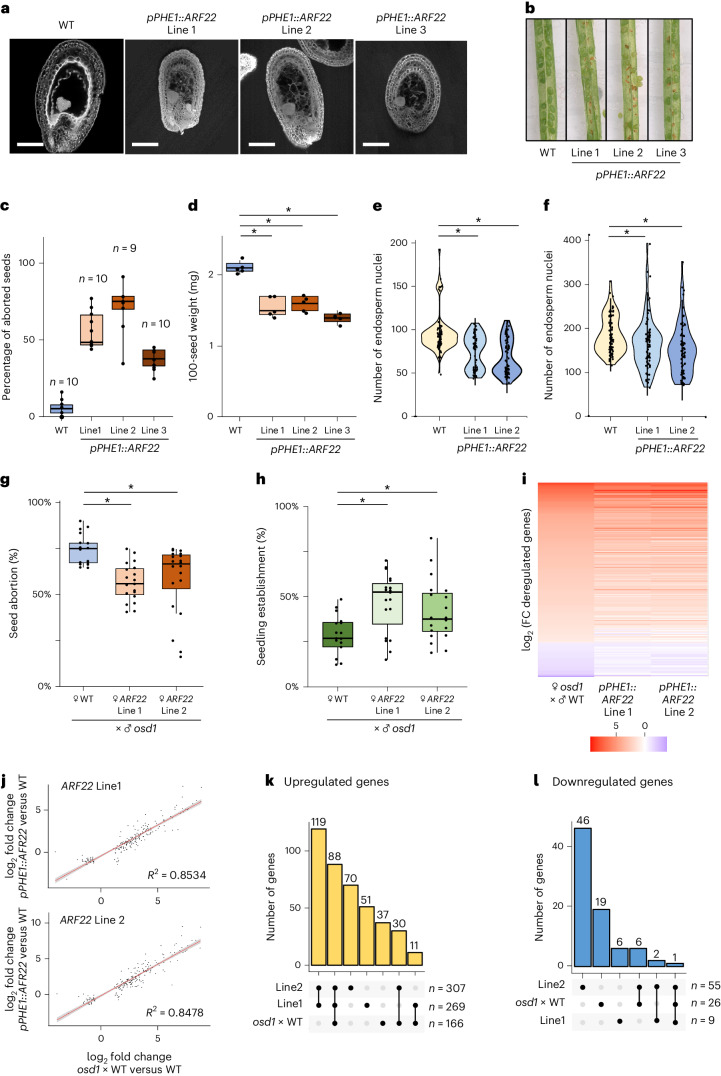
Precocious *cARF* expression promotes endosperm cellularization. **a**, Multiphoton microscopy pictures of 5 DAP Feulgen-stained seeds of 3 independent *pPHE1::ARF22* lines. Scale bars, 100 µm. **b**, Pictures showing seed abortion in the *pPHE1::ARF22* lines. **c**, Quantification of seed abortion in 3 independent *pPHE1::ARF22* lines. Each dot represents the percentage of aborted seeds in one silique. The number of analysed siliques is indicated on the top of boxes. Lines 1 and 2 are hemizygous for the transgene, while Line 3 is homozygous. **d**, The 100-seed weight of seeds from indicated crosses. Each dot represents the weight of 100 seeds. Five independent measurements were analysed for each line (*n* = 5). *P_Line1_ = 4.519 × 10^−5^; *P_Line2_ = 1.669 × 10^−5^; *P_Line3_ = 2.769 × 10^−7^ (two-sided Student’s *t*-test with Bonferroni correction). Boxes show median values and the interquartile range. Whiskers show minimum and maximum values, excluding outliers. **e**,**f**, Endosperm nuclei counts of 3 DAP (**e**) or 4 DAP (**f**) seeds in the *pPHE1::ARF22* lines. Each dot represents the number of endosperm nuclei of one seed. Two biological replicates with more than 30 seeds per replicate were analysed. **e**, *P_Line1_ = 2.129 × 10^−06^; *P_Line2_ = 9.543 × 10^−12^. **f**, *P_Line1_= 0.004038; *P_Line2_ = 7.291 × 10^−06^ (Wilcoxon signed-rank test with Bonferroni correction). **g**, Percentage of aborted seeds derived from indicated crosses. **h**, Percentage of established seedlings from seeds of the indicated crosses. Each dot represents the percentage of aborted seeds (**g**) or established seedlings (**h**) from 3–5 siliques. Five biological replicates were generated, each comprising 3 or 4 inflorescences, resulting in a total of ~18 values. **g**, *P_Line1_ = 2.9666 × 10^−6^; *P_Line2_ = 2.904 × 10^−3^. **h**, *P_Line1_ = 4.858 × 10^−4^; *P_Line2_ = 6.326 × 10^−3^ (Student’s *t*-test with Bonferroni correction). Boxes show median values and the interquartile range. Whiskers show minimum and maximum values, excluding outliers. **i**, Heat map showing the log_2_ FC of deregulated genes in 4 DAP seeds of *osd1* × WT compared to WT, and *pPHE1::ARF22* compared to WT. Only genes that were significantly deregulated in *osd1* × WT compared to WT after multiple-testing correction (|log_2_ FC| ≥ 1; *P*_adj_ < 0.05) are shown. **j**, Correlation plot of log_2_ FCs of deregulated genes in *pPHE1::ARF22* lines and the *osd1* × WT crosses. The linear regression is shown in red and the coefficient of correlation *R*² is indicated in the chart. **k**,**l**, Upset plots showing the number of commonly upregulated (**k**) and downregulated (**l**) genes in the different transcriptomes.

**Extended Data Fig. 6:**
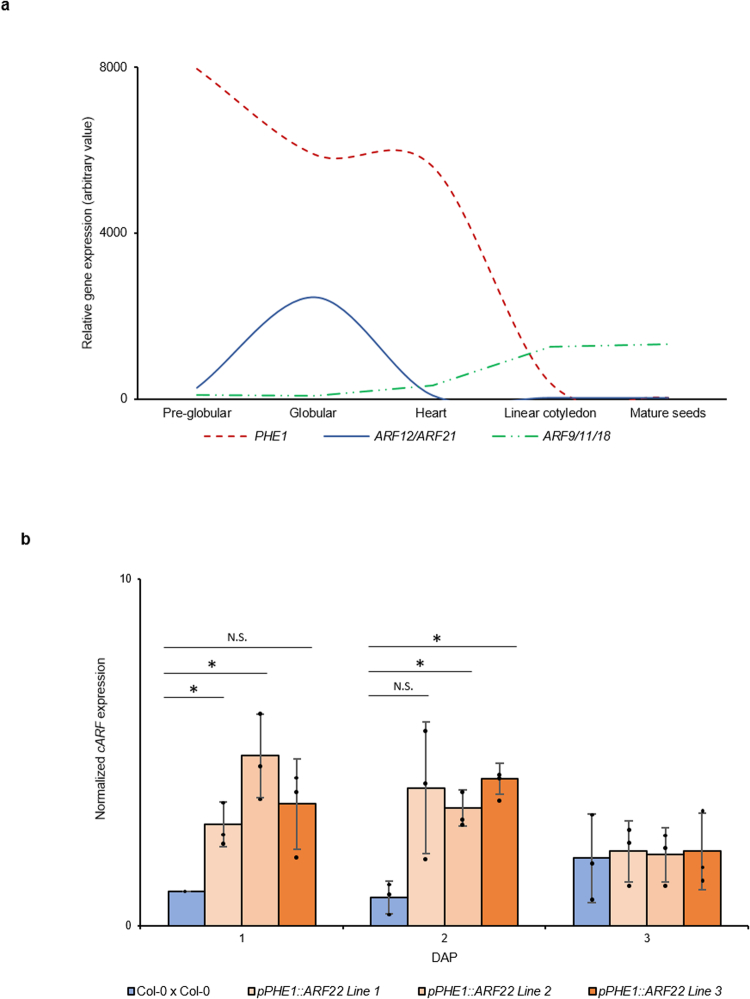
Comparison of relative *PHE1* and *cARF* expression during different stages of endosperm development. (**a**) Expression of *PHE1*, *ARF9/11/18* and *cARFs* based on published ATH1 microarray data^8^. Only *cARF12* and *21* are present on the ATH1 array. (**b**) RT-qPCR analysis of *cARF* expression in 1-, 2- and 3-DAP siliques of the indicated crosses. Data show the mean of three independent biological replicates (n = 3), with error bars indicating the standard deviation. Asterisks denote statistically significant differences based on a two-sided Student’s t-test (*P_1DAP-Line1_ = 6.127 × 10-3; *P_1DAP-Line2_ = 4.864 × 10-3; *P_2DAP-Line2_ = 3.174 × 10-3; *P_2DAP-Line3_ = 8.07 × 10-4). N.S. Not significant.

**Extended Data Fig. 7:**
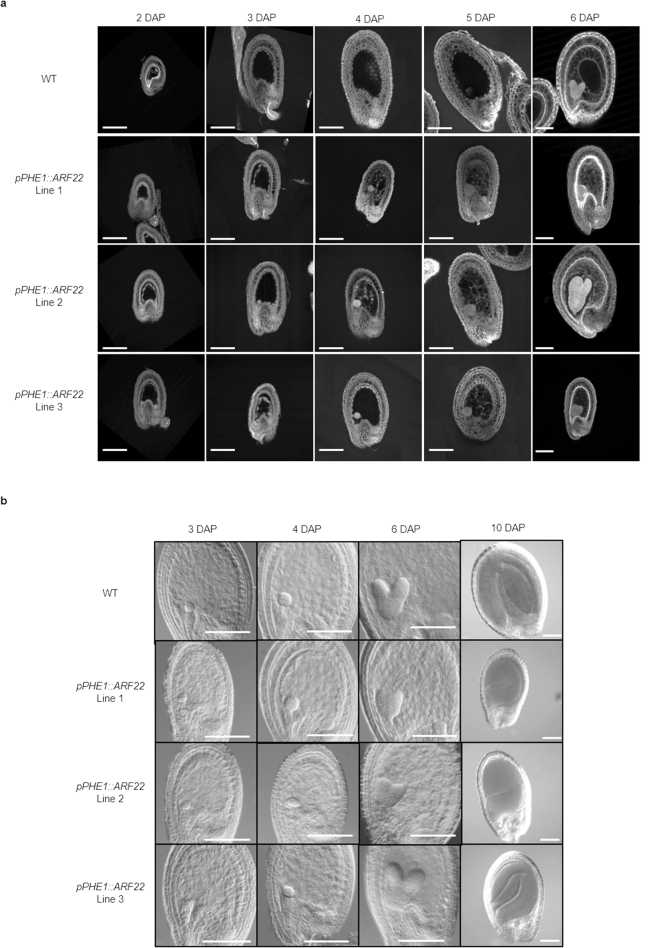
Seed phenotypes of the *pPHE1::ARF22* lines. (**a**) Multiphoton microscopy pictures of Feulgen stained seeds taken at the indicated time points. Data are based on two biological replicates and a minimum of 30 seeds were analyzed per replicate. Scale bars, 100 µm. (**b**) Pictures of cleared seeds taken at the indicated time points. Data are based on two biological replicates and a minimum of 30 seeds were analyzed per replicate. Scale bars, 100 µm.

We next addressed the question of whether precocious expression of *cARFs* is sufficient to induce early cellularization and thus mimic a maternal excess seed phenotype. To this end, we expressed *ARF22* in the endosperm under control of the *PHERES1* (also known as *PHE1*) promoter that is active directly after fertilization and lasts until completion of endosperm cellularization (Extended Data Fig. 6a). Under control of the *PHE1* promoter, *cARFs* were overexpressed at 1 and 2 DAP (Extended Data Fig. 6b). Consistent with the idea that cARFs are required to induce endosperm cellularization, *pPHE1::ARF22* lines produced seeds with precociously cellularized endosperm, preceding wild-type seeds by 1 or even 2 days (Fig. 3a and Extended Data Fig. 7a). Precocious endosperm cellularization was associated with reduced nuclei proliferation, resembling the phenotype of maternal excess seeds^12^ (Fig. 3e,f). Hemizygous *pPHE1::ARF22* lines produced aborted seeds at high frequency (40 to 60%, Fig. 3b,c), revealing that precocious expression of *ARF22* is sufficient to trigger seed arrest. Those seeds contained well developed embryos surrounded by a small, cellularized endosperm, similar to maternal excess seeds^13^ (Fig. 3a and Extended Data Fig. 7a,b). The reduced seed size caused an abnormal position of the embryo, possibly causing seed abortion (Extended Data Fig. 7b). Together, these data show that induction of endosperm cellularization correlates with *ARF22* expression.

**Extended Data Fig. 8:**
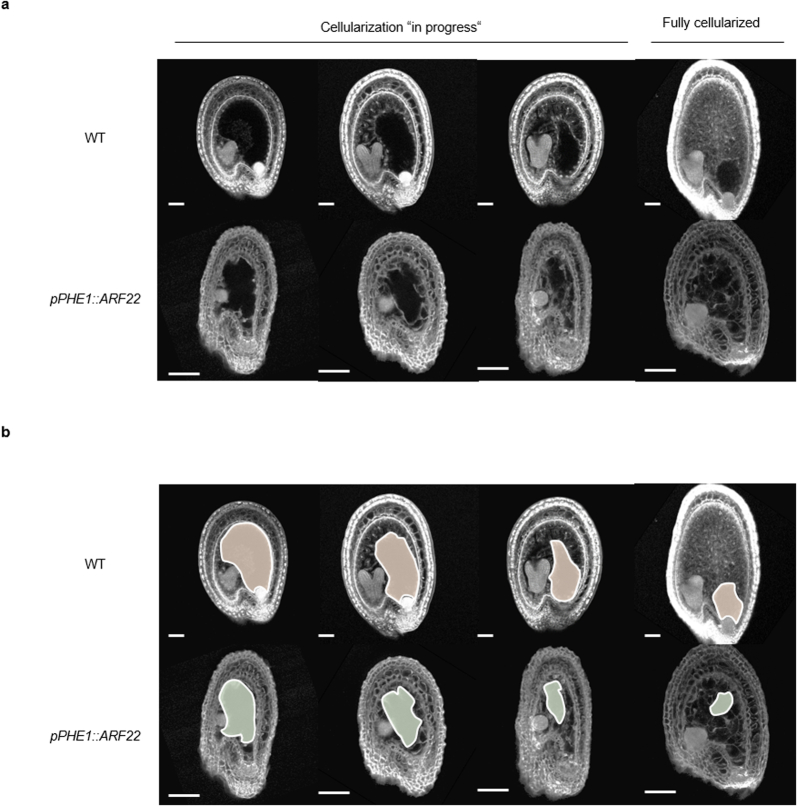
Endosperm cellularization in WT and *pPHE1::ARF22* seeds. (**a**) Multiphoton microscopy pictures of Feulgen stained seeds taken either at 6 DAP (WT) or at 4 DAP (pPHE1::ARF22 seeds). (**b**) Same pictures as in (A) but with the non-cellularized endosperm indicated in brown and green for WT and *pPHE1::ARF22*, respectively. Scale bars, 50um.

**Extended Data Fig. 9:**
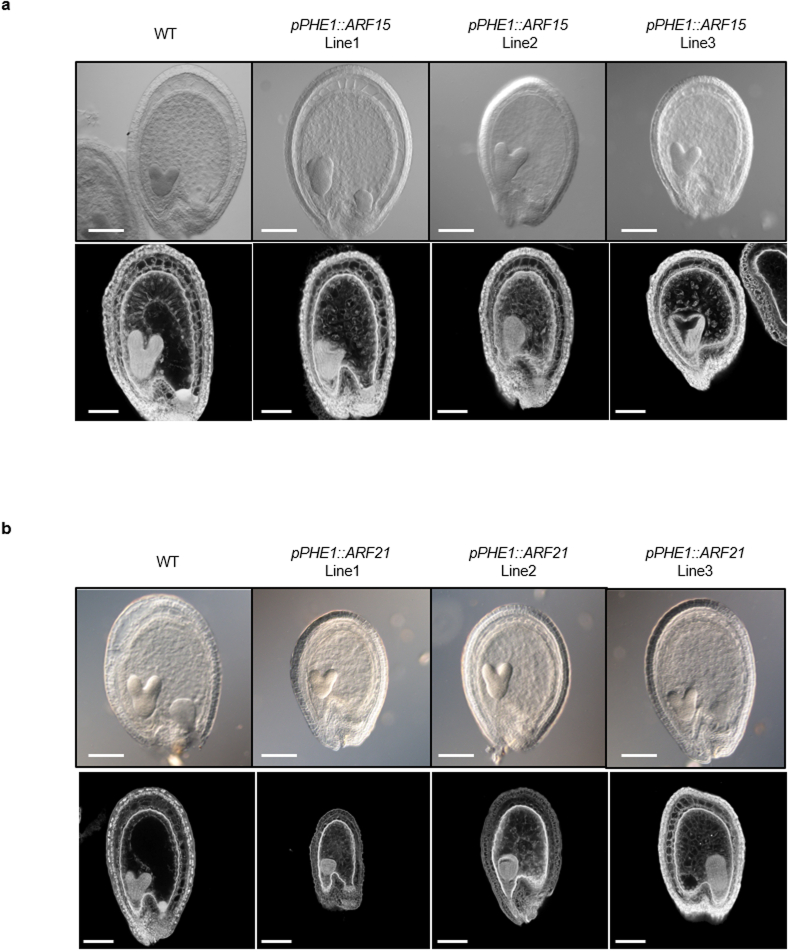
*pPHE1::ARF15* and *pPHE1::ARF21* exhibit an early endosperm cellularization phenotype. (**a,b**) DIC pictures of cleared seeds (upper part) or multiphoton microscopy pictures of Feulgen stained seeds (bottom part), of different *pPHE1::cARF15* and *pPHE1*::*cARF21* (**b**) lines at 6 DAP. Three independent lines were analyzed for each construct. Scale bars, 100 µm.

Interestingly, expression of *pPHE1::ARF22* did not only change the time of endosperm cellularization, but also affected the pattern of this process. In wild-type seeds, endosperm cellularization starts at the micropylar region surrounding the embryo and spreads from there over the whole endosperm^14^ (Extended Data Fig. 8). In contrast, in *pPHE1::ARF22* lines, cellularization started at both ends simultaneously and the generally uncellularized chalazal endosperm became completely cellularized (Extended Data Figs. 7a and 9). This cellularization pattern corresponds with the activity of the *PHE1* promoter, which is strongly expressed in the chalazal region of the endosperm^15^.

Together, these data strongly support the hypothesis that ARF22 directly induces endosperm cellularization.

Similar phenotypes were observed when overexpressing *ARF15* and *ARF21* under control of the *PHE1* promoter, in line with the proposed redundant function of cARFs in promoting endosperm cellularization (Extended Data Fig. 9).

Paternal excess seeds fail to undergo endosperm cellularization, a phenotype which correlated with reduced *cARF* expression (Fig. 1e,f) and that was enhanced by maternal *arf13 arf20* mutants (Fig. 2e). We thus tested whether early cellularization induced by *pPHE1::ARF22* could suppress paternal excess seed lethality. We found a significantly reduced rate of seed abortion when hemizygous *pPHE1::ARF22* lines were pollinated with diploid *osd1* pollen, correlating with increased numbers of viable triploid seedlings (Fig. 3g,h). The increase was nevertheless relatively weak, since overexpression of *ARF22* caused seed lethality at high frequency (Fig. 3b,c).

To test whether the phenotypic similarities between seeds overexpressing *cARFs* and maternal excess seeds was reflected at the molecular level, we compared the transcriptomes of two *pPHE1::ARF22* lines with maternal excess seeds (*osd1* × WT) at 4 DAP. At this timepoint, cellularization had not yet started in WT, but was completed in the other genotypes (Fig. 3a). Significantly deregulated genes (|log_2_ FC| ≥ 1; *P*_adj_ < 0.05) in maternal excess seeds were similarly deregulated in seeds of *pPHE1::ARF22* lines, corresponding to a strong correlation between the datasets (Fig. 3i,j and Supplementary Data 2). The majority (78%) of upregulated genes in maternal excess seeds were also upregulated in at least one of the *pPHE1::ARF22* lines and about half (53%) of them were commonly upregulated in both lines (Fig. 3k,l). The 88 commonly upregulated genes were enriched for functions related to phragmoplast and cytoskeleton fibre formation, consistent with the induced cellularization process (*P* < 0.05).

Together, our data uncover cARFs as key regulators of endosperm cellularization that act in a dosage-dependent manner and probably underpin the parental dosage sensitivity of endosperm cellularization.

### Evolution of *cARFs* in angiosperms

**Extended Data Fig. 10:**
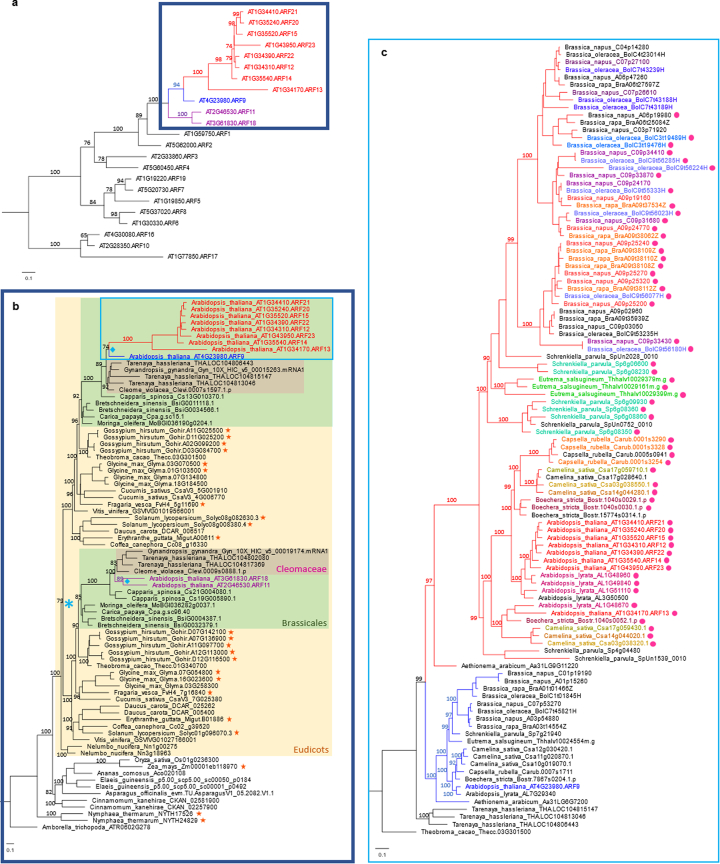
Maximum-likelihood (ML) trees of *ARFs* with bootstrap values supporting branches of interest. (**a**). The phylogeny of 23 *ARFs* in Arabidopsis showing the clade of *cARFs* (red), *ARF9* (blue) and *ARF11/18* (purple). (**b**). The phylogeny of *cARFs* (red), *ARF9* (blue) and *ARF11/18* (purple) in angiosperms. The asterisk marks the eudicot γ- Whole Genome Triplication, and the diamonds mark the Brassicaceae-specific gene duplication. Genes labelled by orange stars are the *ARF9/11/18* homologs with confirmed expression in early-stage endosperm or seed transcriptomes. (**c**). The phylogeny of *cARFs* (red) and *ARF9* (blue) in the Brassicaceae. *cARFs* are colored by tandem clusters. Pink dots label *ARFs* located in pericentromeric regions. The source of sequences, transcriptomes and centromere locations are listed in Supplementary Data 3.

Phylogenetic analysis revealed that *Arabidopsis* *cARFs* are derived from a Brassicaceae-specific duplication of *ARF9* (Extended Data Fig. 10a,b and Supplementary Data 3), and in many Brassicaceae crown species, the ancestral *cARFs* duplicated into tandem arrays nested in pericentromeric regions (Extended Data Fig. 10c). The recurring copy number increase of *cARFs* and the conserved location in pericentromeric heterochromatin suggest selection towards increased maternal-specific expression of *cARFs* in the Brassicaceae.

The *cARFs* are more similar to the tandem paralogues within a species than to orthologues in sister species (Extended Data Fig. 10c), suggesting that frequent events of gene conversion homogenized the cluster of *cARFs*^16,17^. Concerted evolution of *cARFs* leading to multiple copies of nearly identical *cARF* genes may have evolved as a mechanism allowing maternal control of endosperm cellularization. This evolutionary pattern is consistent with the predictions of the parental conflict theory^18,19^, which forecasts the evolution of maternally expressed suppressors of endosperm growth to counteract paternally expressed growth promoters^20^.

The *ARF9* clade arose from the γ-whole-genome triplication shared by all core eudicots^19^, while the paralogous clade corresponds to *ARF11/18* (ref. ^19^) (Extended Data Fig. 10b). The identified orthologue of *ARF9/11/18* in maize, *ZmARF7* (Zm00001eb118970), is expressed in the endosperm sharply around the cellularization stage, putatively promoting the transition from the nuclear to the cellular phase^21^. We thus speculate that the repressive *ARF* clade harbouring the *cARFs* and *ARF9/11/18* play a conserved role in promoting endosperm cellularization. In line with this hypothesis, the orthologues of *ARF9/11/18* in several species are also expressed in the early endosperm or seed transcriptomes (Extended Data Fig. 10b). In contrast, *Arabidopsis* *ARF9/11/18* are not expressed in the early endosperm (Extended Data Fig. 6A), suggesting that the rise of *cARFs* allowed them to adopt specialized functions in the endosperm. The loss of a broad expression pattern may have promoted the increase in copy number without detrimental effects on sporophyte development.

## Discussion

**Fig. 4:**
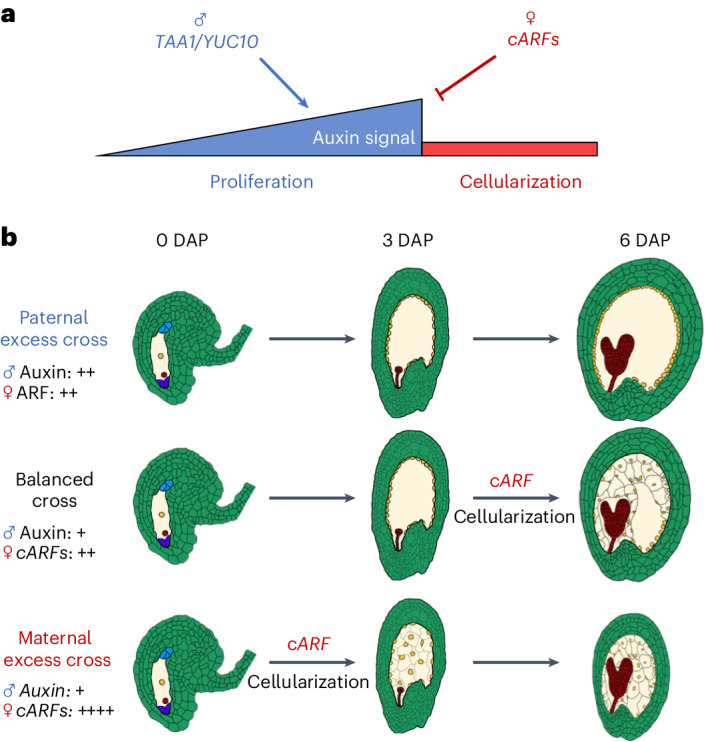
Model depicting antagonistic parental effects on endosperm cellularization via regulation of auxin production and signalling. **a**, After fertilization, the paternally expressed genes *YUC10* and *TAA1* trigger auxin production and initiate endosperm proliferation. Proliferation ends when *cARFs* are expressed from the maternal genome and probably block auxin signalling, thereby inducing endosperm cellularization. **b**, Altering the parental genome dosage changes the time of *cARF* accumulation and endosperm cellularization. In paternal excess crosses, the double dosage of the paternal genome stimulates auxin production, reducing the effect of maternally produced *cARF* transcripts, leading to a delay in or absence of endosperm cellularization. Conversely, in maternal excess crosses, doubling of the maternal genome causes increased accumulation of *cARF* transcripts, precociously reaching the threshold to induce cellularization.

The timing of endosperm cellularization is decisive for final seed size and a major target of parental conflict^22^. Our study reveals that parental-dosage-dependent regulation of *cARFs* controls endosperm cellularization, implicating cARFs as molecular targets of parental conflict (Figs. 2–4).

cARFs belong to the evolutionarily conserved ARF B class that are considered to be transcriptional repressors^23,24^. Repressive B class ARFs were shown to antagonize activating A class ARFs^25^, providing an intuitive model whereby cARFs block auxin-mediated endosperm proliferation^4^ by competing with activating A-type ARFs that remain to be identified (Fig. 4a). In support of this view, we found that increased dosage of cARFs reduced endosperm proliferation (Fig. 3e,f).

A key prediction of the parental conflict theory is that maternal and paternal genomes antagonistically affect the growth of embryo supportive tissues^20^. Specifically, natural selection is expected to favour paternally active alleles promoting seed growth and maternally active alleles restricting seed growth. By promoting endosperm cellularization and thus restricting seed growth, cARFs are probably major targets of this conflict. Consistent with the predictions regarding maternally biased expression of growth suppressors, *cARFs* are maternally expressed while paternally silenced by a combination of repressive epigenetic modifications (Fig. 1c,d and Extended Data Fig. 2b–d). Interestingly, within the Brassicaceae, we found evidence for a repeated amplification of *cARFs* into tandem arrays nested in pericentromeric regions (Extended Data Fig. 10b). This recurring copy number increase of *cARFs* is probably a consequence of parental conflict, ensuring maternal control of endosperm cellularization. Auxin biosynthesis in the endosperm is controlled by the paternal genome and increased auxin levels delay endosperm cellularization^5^, revealing an antagonistic parental control of endosperm cellularization converging on auxin biosynthesis and signalling (Fig. 4b).

In conclusion, we identified cARFs as maternally active dosage-sensitive regulators of endosperm cellularization. cARFs induce endosperm cellularization and thus restrict seed growth, making them direct molecular targets of parental conflict in angiosperm seeds.

## Methods

### Plant cultivation and lines used in this study

The *Arabidopsis* mutant *osd1-3* has been previously characterized^11^. The *arf15-1* (SALK_029838) and *arf20-2* (SALK_032522) mutants have been published^6^. The *arf22-3* (SALKseq_49790) mutant has been characterized in this study. Primers used to genotype the mutants are listed in Supplementary Data 4. For all experiments, Col-0 was used as the wild-type control.

*Arabidopsis* seeds were sterilized for 15 min in a solution of 70% ethanol and 0.0001% Triton X-100 and washed with 100% ethanol for an additional 15 min. Dried seeds were sown on plates containing ½ Murashige and Skoog medium and stratified at 4 °C for 2 days. Plates were incubated in a growth chamber for 2 weeks (16 h light/8 h dark, 60 µmol s^−1^ m^−2,^ 22 °C), then transferred to soil and grown in phytotron chambers (16 h light/8 h dark, 150 µmol s^−1^ m^−2^, 21 °C, 70% humidity).

### Generation of plasmids and transgenic plants

Genes were amplified from *Arabidopsis* Col-0 genomic DNA with the primers described in Supplementary Data 4. After amplification, the fragments were inserted into a pENTR vector by using the pENTR/D-TOPO kit (ThemoFisher, K240020SP). For the *pPHE1::cARFs*, the fragments were inserted into the pPHE1-pB7WG2 (ref. ^4^) vector using an LR reaction (ThermoFisher, 11791020). For the *pARF15::ARF15-GFP* and *pARF22::ARF22-GFP* constructs, the destination vector was pB7FWG.0.

For *pARF13::ARF13* and *pARF20::ARF20*, the amplified fragments were first introduced in a pDONR221 vector using a BP reaction (ThemoFisher, 11789100). The fragments were then inserted into a pBGW0 vector using an LR reaction (ThermoFisher, 11791020).

For the CRISPR construct, the guide RNA sequences for mutating cARFs were designed by E-CRISP4. Two guide RNAs were chosen to target cARF genomic DNA: DT1(AAGTTTATTACTTTCCTCAAGGG) and DT2c (AAAGATCCCATTGAAGAAATTGG). The construction protocol has been previously published^26,27^. The PCR fragment was amplified from pCBC-DT1T2 with the four primers listed in Supplementary Data 4 and inserted into pHEE401E by Golden Gate cloning.

All constructs were introduced into the *Arabidopsis* Col-0 accession using the floral dip protocol^28^. Transformed plants were selected on medium containing appropriate chemicals.

### Microscopy

For monitoring *pARF15::ARF15-GFP* and *pARF22::ARF22-GFP*, siliques were opened at the indicated stage and seeds were mounted in water. Fluorescence was observed using a LEICA Stellaris 8 Dive microscope with an excitation of 488 nm and an emission range of 493–551 nm. The data were generated by first analysing a minimum of 15 seeds to determine whether a signal is present or not. If a signal was detected, we observed at least 40 and recorded only the average phenotype excluding any atypical signal.

For clearing and Feulgen staining, siliques were opened at indicated stages and incubated overnight at 4 °C in a fixing solution of ethanol:acetic acid (3:1). On the next day, the solution was replaced with 70% ethanol and stored at −20 °C until staining.

For seed clearing, the seeds were removed from the siliques and incubated overnight at 4 °C in a clearing solution (66.7% w/w chloralhydrate, 8.3% w/w glycerol). They were then mounted in clearing solution and observed on an Olympus BX-51 microscope.

Sample preparation and embedding for Feulgen staining were done as previously described^3^. Samples were observed on a LEICA Stellaris 8 Dive microscope using the multiphoton mode with an excitation of 800 nm and an emission range of 563–668 nm.

Endosperm nuclei, aborted seeds and seedling establishment were counted using the Fiji software.

### RNA extraction, RT–qPCR and library preparation

For RT–qPCR, two siliques were harvested at the indicated stage, ground in liquid nitrogen and stored at −80 °C until extraction. For mRNA sequencing, ~500 seeds were dissected from siliques and stored in RNAlater solution (ThermoFisher, AM7021) at 4 °C before extraction.

RNA was extracted using the RNeasy plant mini kit (Qiagen, 74904). RNAs were treated with DNAseI at 37 °C for 30 min (ThermoFisher, EN0521). DNAseI was inactivated by incubation at 65 °C for 10 min and removed by TRIzol extraction before library construction following the manufacturer’s protocol (ThermoFisher, 15596018).

The reverse transcription reaction was performed using the RevertAid H Minus First Strand cDNA Synthesis kit (ThermoFisher, K1631) and a dTTTN primer (Supplementary Data 4). The qPCR was performed with the Power SYBR Green PCR Master Mix (ThermoFisher, 4367659) and the indicated primers (Supplementary Data 4). The efficiency for the GAPDH primers was 99.6% and 100% for the cARFs. The relative quantification of the cARF expression normalized to GAPDH was calculated as defined by the Bio-Rad qPCR manual.

The mRNA libraries were generated using the NEBNext Ultra II DNA Library Prep kit (NEB, E7645S) coupled to the NEBNext Poly(A) mRNA Magnetic Isolation Module (NEB, E7490S). Sequencing was done by Novogene on a HiSeqX in 150-bp paired-end mode.

### RNA-seq analysis

For each replicate, 150-bp-long paired-end reads were trimmed using Trimgalore (5 bp at the 5′ end and 20 bp at the 3′ end) and mapped to the *Arabidopsis* (TAIR10) genome using hisat2. Mapped reads were counted using Htseq-count and normalized to transcripts per million (TPM) for genes using StringTie. Differentially regulated genes between conditions and across the replicates were detected using DESeq2 applying a threshold of log_2_ FC ≥ 1 with a false discovery rate adjusted *P* value of <0.05. Non-metric multidimensional scaling (NMDS) multivariate analysis was performed to assess the replicability and degree of similarity between samples using the metaMDS function of the vegan package in R. NMDS is a non-parametric ordination method where the dissimilarity distances among all pairs of samples are ranked. Dissimilarities were calculated using the Bray–Curtis index applied to gene expression values (TPM). Charts were generated using the R package ggplot2 and Microsoft Excel 2019.

### Phylogenetic analyses

To elucidate the relatedness within the ARF family, amino acid sequences of all 23 ARFs in *Arabidopsis* were obtained from TAIR10. MUSCLE was used to generate the multiple sequence alignments with default settings^29^. The sequences of the three defining functional domains: B3 type DNA-binding domain (InterPro, IPR003340), auxin response factor domain (IPR010525) and AUX/IAA domain (IPR033389), were identified by the conserved domain search tool, CD-Search^30^, and were extracted and aligned independently to generate the concatenated alignments of conserved ARF protein regions. IQ-TREE 1.6.7 was applied for maximum-likelihood inference of the phylogeny^31^, with the JTT substitution model as suggested by the implemented ModelFinder^32^ and 1,000 ultrafast bootstrap replicates to estimate the support for reconstructed branches^33^. The phylogenetic tree figure was generated by Figtree.

To analyse the phylogenetic timing of *cARF* and *ARF9* duplication, amino acid sequences of homologues of ARF9, ARF11 and ARF18 were identified in several angiosperm species, with an emphasis on Brassicales (Supplementary Data 3). Full-length sequence alignments using MUSCLE were used as input for the IQ-TREE analyses, following the procedure above.

To investigate the pattern of *cARF* evolution after the divergence from *ARF9*, amino acid sequences and nucleotide sequences of cARFs and ARF9 in several Brassicaceae species (Supplementary Data 3) were used to generate a guided codon alignment in MUSCLE. A maximum-likelihood tree was then generated in IQ-TREE with the codon alignment as input, and using the GTR substitution model and 1,000 replicates of ultrafast bootstrap.

### Reporting summary

Further information on research design is available in the Nature Portfolio Reporting Summary linked to this article.

### Supplementary information

Supplementary Information Alignment *cARF* coding sequence using CLUSTAL omega. The protein domains were annotated on the basis of TAIR annotation.

Reporting Summary

Supplementary Data 1 Alignment *cARF* coding sequence using CLUSTAL omega. The protein domains were annotated on the basis of TAIR annotation.

Supplementary Data 2 (A) Table showing normalized reads for 7 DAP seed transcriptomes. (B) and (C) Tables showing DESeq2 results comparing WT transcriptomes to Col-0 × *osd1* (B) or to *arf13 arf20* × Col-0 (C).

Supplementary Data 3 (A) Table showing normalized reads for 4 DAP seed transcriptomes. (B)–(D) Tables showing DESeq2 results comparing WT libraries to *osd1* × Col-0 (B), to *pPHE1::ARF22* Line1 (C) or to *pPHE1::ARF22* Line 2 (D).

Supplementary Data 4 List and functions of the primers used in this study.

## Data Availability

RNA-seq data generated in this study are available at NCBI’s Gene Expression Omnibus database under the accession number GSE232803. The imprinting, CHiP-seq, DNA methylation and endosperm expression data can be found under GSE66585 (ref. ^34^), GSE84122 (ref. ^35^), GSE12404 (ref. ^8^) and GSE157145 (ref. ^36^), respectively. Sequence analysis was based on the *Arabidopsis* TAIR10 genome.
