# Supplementary material for: Parental conflict driven regulation of endosperm cellularization by a family of Auxin Response Factors
Source: Nat Plants. 2024 May 28;10(6):1018–26. doi: 10.1038/s41477-024-01706-y (PMC11208147; doi:10.1038/s41477-024-01706-y)
Supplement: Supplementary file 1 — Alignment cARF coding sequence using CLUSTAL omega. The protein domains were annotated on the basis of TAIR annotation. [file 41477_2024_1706_MOESM1_ESM.pdf]

nature plants

Supplementary information https://doi.org/10.1038/s41477-024-01706-y

Parental conflict driven regulation of endosperm cellularization by a family of Auxin Response Factors

In the format provided by the authors and unedited

Supplementary information: Sequence alignment of cARFs.

```
CLUSTAL O(1.2.4) multiple sequence alignment

ARF13           ATGGAAAATAATGGAGAAATGAATGCACAGCCTGAATTATCAGgcataagtaaagatttt 60
ARF14           ATGGAAAGTGGCAACGTTGTGAATACACAACCTGAATTATCAGGCATAAgtaagaaattt 60
ARF22           ATGGAAAGTGGCAACATTGTGAATGCACAACCTGAATTATCAGGCATAAgtaagaaattt 60
ARF12           ATGGAAAGTGGCAACGTTGTGAATGCACAACCTGAATTATCAGGCATAAgtaagaaattt 60
ARF20           ATGGAAACTGGCAACGTTGTGAATGCACAACCTGAATTATCAGGCATAAgtaagaaattt 60
ARF15           ATGGAAACTGGCAACGTTGTGAATGCACAACCTGAATTATCAGGCATAAgtaagaaattt 60
ARF21           ATGGAAAGTGGCAACATTGTGAATGCACAACCTAAATTATCAGGCATAAgtaagaaattt 60
                ******* *          ***** **** *** *******************  * ***

ARF13           tttttatatatattttgtctgaacttattttatatattaaatatgaaacaaccatatatt 120
ARF14           tgattt------ttttattttat----------tgaaaacttgttttagttataatgttt 104
ARF22           tgacttttt--attttattttag----------tgaaaacttgttttggttataatgttt 108
ARF12           taacttttt--attttattttat----------tgaaaacttgttttggttataa-tttt 107
ARF20           taactttt-ttattttctcttat----------tgaaaacttgttttggttataattttt 109
ARF15           tgactttttttattttatcttat----------tgaacacttgttttggttataatgttt 110
ARF21           tgactttt--tattttattttat----------tgaaaacttgttttggttataatgttt 108
                *   *       **** * * *             *  *  * *         *    **

ARF13           ttgtttcgttttactagTTGATATAACCAAGACTTATATGTATGAGAAATTATGGAACAT 180
ARF14           tgatttttgttcggcagTTGATGGATCCAAGAGTTATATGTACGAGCAGTTATGGAAACT 164
ARF22           tagtttttgttcggcagTTGATGGATCCAAGAGTTATATGTACGAGCAGTTATGGAAACT 168
ARF12           tagtttttttccggtagTTGATGGATCCAAGAGCTATGTTTACGAGCAGTTATGGAAACT 167
ARF20           tggtttttgttcggcagTTGATGGATCCAAGAGCTATATGTACGAGCAGTTATGGAAACT 169
ARF15           tggtttttgttcggcagTTGATAGATCCAAGAGCTATATGTACGAGCAGTTATGGAAACT 170
ARF21           tagtttttgttcggcagTTGATGGATCCAAGAGCTATATGTACGAGCAGTTATGGAAACT 168
                *  ***   *     *******  * ******  *** * ** *** * ********  *

ARF13           ATGTGCTGGACCTTTGTGTGTTCTTCCGAAACCTGGAGAAAAAGTTTATTACTTTCCTCA 240
ARF14           CTGTGCGGGACCTTTGTGTGATATTCCAAAACTTGGAGAAAAGGTTTATTACTTTCCTCA 224
ARF22           CTGTGCGGGGCCTTTGTGTGATATTCCAAAACTTGGAGAAAAGATTTATTACTTTCCTCA 228
ARF12           CTGTGCGGGACCTTTGTGTGATATTCCAAAACTTGGAGAAAAGGTTTATTACTTTCCTCA 227
ARF20           CTGTGCGGGACCTTTGTGTGATATTCCAAAACTTGGAGAAAATGTTTATTACTTTCCTCA 229
ARF15           CTGTGCGGGACCTTTGTGTGATATTCCAAAACTTGGAGAAAAGGTTTATTACTTTCCTCA 230
ARF21           CTGTGCAGGGCCTTTGTGTGATATTCCAAAACTTGGAGAAAATGTTTATTACTTTCCTCA 228
                 ***** ** ********** * **** **** *********  ****************

ARF13           AGGGCACATCGAGCTCgtgagtctatatatatttcttttgttttgc-------------- 286
ARF14           AGGCCATATAGAGCTTgtgagttctctctctctatattctttctctcttctttctctctc 284
ARF22           AGGCAATATAGAGCTTgtgagttctctctctttttttttttttttttttttttctctctctc 288
ARF12           AGGTCACATAGAGCTTgtgagttctctctctctctctctctccctctcttttc--------- 278
ARF20           AGGCAATATAGAGCTTgtgagttctccctctcttctct-------------------ctc 270
ARF15           AGGCAATATAGAGCTTgtgagttctctctctctctctctctcctc--------------- 275
ARF21           AGGCAATATAGAGCTTgtgagttctctctctttttgtttctctctctctctctctctctc 288
                ***  * ** ***** ******          *

ARF13           ------------------------------------------------------------ 286
ARF14           tctctctctctctctctct----------ctctctctctctctctctctctctctctctc 334
ARF22           tctctctcttctttctctctccctctcttctctctctctctctctctctctctctttctt 348
ARF12           --tttctctc---------tcca------------------------------------- 290
ARF20           tctctctctc---------tccctctcttctctctctcttc-------tctttctctctt 314
ARF15           --tctctctc---------tccctctc-----------ttc-------tctttct--ctc 304
ARF21           tctctctctc---------tccctctcttctctctctcttc-------tctttctctctt 332


ARF13           ------------------------------------------------------------ 286
ARF14           ctt------tctctctcttctctctcttctctctctcgcctctttctctctctctctctc 388
ARF22           ctttctctctccctctcttctctccctcttctctctctctctcttctttctccctatctc 408
ARF12           --------------------------tctcttctctctctc------------------- 305
ARF20           ctctctctctttcttctttctatctccctttcttctctctctcttctt------------ 362
ARF15           ttctctctcttctttct--ctctccctttcttctctctc----tctct------------ 346
ARF21           ctctctctcgtctttct--ctctccctttcttctctctctctcttctt------------ 378


ARF13           -------ttctcgatttggtttttgaaacattttgatggcacttttcagATTGAGAATTC 339
```

```
ARF14      tctctctccttctctctcatgtatgaa-accttttgacggctttttcagGTTGAGGCATC 447
ARF22      tctctccctctctctcgtgtatcaaaa-ccttttgacgactttttcagGTTGAGGCATC 467
ARF12      ----tcttctctctctctctgtatgaa-accttttaacggggttttcagGTTGAGACATC 360
ARF20      tctctctatctctctcttgtgtatgaa-accttttgacggccttttcagGTTGATGCATC 421
ARF15      cttctctctctctctctcgtctatgaa-accttttgacggctttttcagGTTGAGGCATC 405
ARF21      tctctctatctttctctcgtgtatgaa-accttttgacggccttttcagGTTCAGGCATC 437
                              * *   **    ***        ******* ** *    **

ARF13      TACAAGAGATGAATTGGATCATATCAGGCCAATTTTTGATCTTCCATCTAAGCTTCGATG 399
ARF14      TACAAGAGAAGAGTTGAATGAGTTACAACCAATTTGTGATTTTCCTTCTAAGCTTCAATG 507
ARF22      TACAAGAGAAGAGTTGAATGAGTTAAAACCAATTTGTGATCTTCCTTCTAAGCTTCAATG 527
ARF12      TACAAGAGAAGAGTTGAATGAGTTACAACCAATTTGTGATCTTCCTTCTAAGCTTCAATG 420
ARF20      TACAAGAGAAGAGTTGAATGAGTTACAACCAATTTGTGATCTTCCTTCTAAGCTTCAATG 481
ARF15      TACAAGAGAAGAGTTGAATGAGTTACAACCAATTTGTGATCTTCCTTCTAAGCTTCAATG 465
ARF21      TACAAGAGAAGAGTTGAATGAGTTACAACCAATTTGTGATCTTCCTTCTAAGCTTCAATG 497
           ********* ** *** ** *  *    ******* **** **** ********** ***

ARF13      TCGTGTTGTGGCTATTGATCGTAAGgtatgtctttaaatgttcaagagttaacacataag 459
ARF14      TCGTGTTATTGCTATTCAACTTAAGgtatatctctattatgttttagcgaatacgcatga 567
ARF22      TCGTGTTATTGCTATTCAACTTAAGgtatatctctattatgttttaccgaatacgcatga 587
ARF12      TCGTGTTATTGCTATTCATCTTAAGgtatatctctattatgttttagcgaatacgcatga 480
ARF20      CCGTGTTATTGCAATTCATCTTAAGgtatatctctattatgttttagcaaatacgcatga 541
ARF15      TCGTGTTATTGCAATTCATCTTAAGgtatatctctattgtgttttagcgaatacgcatga 525
ARF21      TCGTGTTATTGCAATTCATCTTAAGgtatatctctattatgttttagcgaatacgcatga 557
            ****** * ** *** * * ******** *** **     *        *

ARF13      ttacaatttattatttgcatgctttatttcactgtagaaag-------atctaatttg 512
ARF14      tt--------------tttatagttgatctaattataaacagaaataattggttaattgt 613
ARF22      ta--------------tata--tttgatttcattgtaaaaataagtaattggttaattgt 631
ARF12      ta--------------tgtatatttaatttcattgtaaaaagacgtaattgggtaattgt 526
ARF20      ta--------------tctatatttgatttcattgtaaaaagaagtaattggttaattgt 587
ARF15      ta--------------tctatatttgatttcattgtaaaaagaagtaattagttaatttt 571
ARF21      ta--------------tctatatttgattttattgtaaaaagaagtaattggttaattgt 603
           *               *  *   ** ** * * * ** * *             * **

ARF13      ttgcttgctttatgtttattatagGTAGACAAAAATACAGATGAAGTCTATGCTCAGATT 572
ARF14      ttga-------tgtttgattttagGTGGAAAACAATAGTGATGAGACTTATGCTGAGATC 666
ARF22      ttga-------tgtttgattttagGTGGAAAACAATAGTGATGAGACTTATGCTGAGATC 684
ARF12      ttga-------tgtttgattttagGTGGAAAACAATAGTGATGAGACTTATGCTGAGATC 579
ARF20      ttga-------tgtttgattttagGTGGAAAACAATAGTGATGAGACTTATGCTGAGATC 640
ARF15      ttga-------tgtttgattttagGTGGAAAACAATAGTGATGAGACTTATGCTAAGATC 624
ARF21      ttgatgtttgatgtttgattatagGTGGAAAACAATAGTGATGAGATTTATGCTGAGATC 663
           ***           ** *** ***** ** ** ****  *****    ****** ****

ARF13      TCGTTAATGCCTGATACAACAgtgagtgaccgcaacaatgctaacttcaaaattaattct 632
ARF14      ACTTTGATGCCAGATACAACTgtaagtgaaaatttagtatttctcttttattt--t--ca 722
ARF22      ACTTTGATGCCAGATACAACTgtaagtgaaaatttagtatttctcttttattt--t--ca 740
ARF12      ACTTTGATGCCAGATACAACTgtaagtgaaaatttagtatttctctt--ttattat--ta 635
ARF20      ACTTTGATGCCAGATACAACTgtaagtgaaaatttagtatttctc--ttttatttt--ca 696
ARF15      ACTTTGATGCCAGATACAACTgtaagtgaaaatttagtatttctc--ttttatttt--ca 680
ARF21      ACTTTGATGCCAGATACAACTgtaagtgaaaatttagtattttctcttttatttt--ca 721
            * ** ***** ******** ** *****            *  *       *

ARF13      aatgtttctcaattgtttcaaatttcttcaattttcacttaactaatttttttaaattg 692
ARF14      gttaattctatttt-tctcc----------tattatatctcattgattttctgattttgt 771
ARF22      gttaattctatttttttctcttatta----------tagctgattgattttctgattttgt 790
ARF12      gttaattctatttt-cctcctattataatacactgat----------tttctgattttgt 684
ARF20      gtcaattgtatttt-tctcctattatatctcattgatttt----------ctgattttgt 745
ARF15      gttaattgtatttt-tctccaattatatctcattgatttt----------ctgattttgt 729
ARF21      gtcaattgtatttt-tctcctattatatctcattgatttctgatttttctgattttgt 780
                ** *   **   **                                *      *

ARF13      taaatctttagGAAGTTATGACCCATAATACTACTATGGATACTCGAAGACCAATAGTTT 752
ARF14      taaatttttagCAAGTTGTAATCCCTACTCAGAATCAAAATCAATTTAGGCCATTAGTTA 831
ARF22      taaatgtttagCAAGTTGTAATCCCTACTCAAAATGAAAATCAATTTAGACCATTAGTTA 850
ARF12      taaatgtttagCAAGTTGTAATCCCTACTCAGAATGAAAATCAATTTAGACCATTAGTTA 744
ARF20      taaatccttagCAAGTTGTAATCCCTACTCAGAGTGAAAATCAATTTAGGCCATTAGTTA 805
ARF15      tgaatgtttagCAAGTTGTAATCCCTACTCAGAATGAAAATCAATTTAGGCCATTAGTTA 789
```

```
ARF21      taaattcttagCAAGTTGTAATCCCTACTCAGAGTGAAAATCGATTTAGGCCATTAGTTA 840
           * ***  **** ***** * * ** ** *   * *    **      ** *** *****

ARF13      ATTTTTTTAGTAAAATTTTAACGGCGTCTGACGTCAGTTTAAGTGGTGGATTAATTATTC 812
ARF14      ATTCCTTTACGAAGGTTTTAACGGCCTCAGATACAAGCGTTCATGGTGGATTCTCTGTTC 891
ARF22      ATTCCTTTACGAAGGTTTTAACGGCCTCGGATACAAGCG------GTGGATTCTTTGTTC 904
ARF12      ATTCCTTTACGAAGGTTTTAACGGCCTCAGATACAAGCGCTCATGGTGGATTCTTTGTAC 804
ARF20      ATTCCTTTACGAAGGTTTTAACGGCCTCTGATACAAGCGCTTATGGTGGATTCTTTGTTC 865
ARF15      ATTCCTTTACAAAGGTTTTAACTGCCTCTGATATAAGTGCTAATGGTGTATTTTCTGTTC 849
ARF21      ATTCCTTTACAAAGGTTTTAACGGCCTCAGATACAAGCGCTTATGGTGGATTCTCTGTTC 900
           ***  ****  **  ******* ** ** **    **        *** ***   * * *

ARF13      CCAAACAATATGCCATTGAGTGTTTTCCTCCGCTGgtaattctgcttagtttatctattg 872
ARF14      CGAAAAAACATGCCATTGAATGTCTCCCTCCGCTGgtatttaatttatagttttt-taa 950
ARF22      CAAAAAAACATGCCATTGAATGTCTTCCTCCCCTGgtatttaatgttatagttttt-taa 963
ARF12      CGAAAAAACATGCCATTGAATGTCTCCCTTCGCTGgtatttaatgttatagtttttttaaa 864
ARF20      CGAAAAAACATGCCATTGAATGTCTCCCTCCACTGgtatttaatgttataattttttttaa 925
ARF15      CGAAAAAACATGCCATTGAATGTCTCCCTCCTCTGgtatttaatgttatagctttt-taa 908
ARF21      CGAAAAAACATGCCATTGAATGTCTCCCTCCGCTGgtatttaatgttatagtttttttaa 960
           * *** ** ********** *** * *** * ****** **    ***     * *

ARF13      aataaactcgaatttgagtttcagtattacattttctattcgatttgtttctctagGATA 932
ARF14      acgaatttgaattttaagctttcattagaca--ttacatattaaatttggtttagGATA 1008
ARF22      acgaatttgaattttaaactttcattagaca--ttacatattaaatttggtttagGATA 1021
ARF12      ataaatttaaactttaatctttcattagaca--ttacatattaaatttggtttagGATA 922
ARF20      atgaatttgaattttaagatttcattagaca--ttacatattaaatttggtttagGATA 983
ARF15      acgaatttgaattttaagctttcattagaca--taacatattaaattttgtttagGATA 966
ARF21      acgaatttgaattttaagctttcattagaca--ttacatattaaatttggtttagGATA 1018
           *  **  *  * *** *  **    *   ***  *   **    *  * **  * *******

ARF13      TGTCCCAACCAATATCCACACAAAATCTTGTTGCAAAGGATCTCTATGGTCAAGAATGGA 992
ARF14      TGTCACAGCCTCTACCGACACAAGAAATTCTTGCTATAGATCTTCATGGTAATCAATGGA 1068
ARF22      TGTCTCAGCCTCTACCGACACAAGAACTTCTTGCTACAGATCTCCATGGTAATCAATGGA 1081
ARF12      TGTCTCAACCTTTACCTGCACAAGAACTTCTTGCTATAGATCTCCATGGTAATCAATGGA 982
ARF20      TGTCTCAGCCTCTACCGGCACAAGAACTTCTTGCTAAAGATCTCCATGGTAATCAATGGA 1043
ARF15      TGTCTCAGCCTCTACCGGCACAAGAACTTCTTGCTATAGATCTCCATGGTAATCAATGGA 1026
ARF21      TGTCTCAGCCTCTACCGGCACAAGAAATTCTTGCTATAGATCTCCATGATAATCAATGGA 1078
           **** ** **  ** *  ***** *  ** **** *  *****  *** * *  ******

ARF13      GTTTCAAACATGTCTTTAGAGgtatttaatatttttcttcacccgataat---------- 1042
ARF14      GATTCAGACACATCTATAGAGgtaataattccttttatt-atataattaaaacacagcta 1127
ARF22      GATTCAACCACAACTATAGAGgtaataattccttttattcatataattcaaacacaacta 1141
ARF12      GATTCAACCACAACTATAGAGgtaatgattccttttattcatataattcaaacacaacaa 1042
ARF20      GATTCAGACACAGCTATAGAGgtaataattcattttattcaaatcattcaaacacagcta 1103
ARF15      GCTTCAGACACAGCTATAGAGgtaataattcattttattcaaataattcaaccacagcta 1086
ARF21      GATTCAGACACAACTATAGAGgtaataattcattttattcaaataattcaaacacaacta 1138
           * ****  **   ** ******** * * *  **** ** *    **

ARF13      -------------attgtaattagtaactatggatcttctttttgcatgtagGTACACCG 1089
ARF14      ttgaggtaataaatcctagagaagtaactaataatcttcttattccatgtagGTACAGCG 1187
ARF22      ttgaggtaataaatcctagagaagtaactaataatcttcttatcccatgcagGTACACCG 1201
ARF12      ttgaggtaataaatcctagataaataactaataatcttcttattccatgcagGTACACCG 1102
ARF20      ttgaggtaataaatcctagagaagtaactaataatcttcttattccatgcagGTACACCG 1163
ARF15      ttgaagtaataaatcctagagaagtaactaataatcttcttattccatgcagGTACACCG 1146
ARF21      ttgaggtaataaatcctatagaagtaactaataatcttcttattccatgaagGTACACCG 1198
                              *  * ******   ******** *  **** ******* **

ARF13      CAGAGACATATGTTTACTAGCGGCGGCGGCTGGAGCGTATTTGCAACAACAAAAAGATTG 1149
ARF14      CAAAGACATTTGTTAACAATTGG------TTGGAATGCATTCACAACATCAAAAAAATTG 1241
ARF22      CAAAGACATTTGTTAACAACTGG------TTGGAATGCATTCACAACATCAAAAAAATTG 1255
ARF12      CAAAGACATTTGTTAACAACTGG------TTGGAATGCATTCACAACATCAAAAAAATTG 1156
ARF20      CAAAGACATTCGTTAACAACTGG------TTGGAATGAATTCACAACATCGAAAAAGTTG 1217
ARF15      CAAAGACATTTATTAACAACTGG------TTGGAATGAATTTACAACATCAAAAAAATTG 1200
ARF21      CAAAGACATTCGTTAACAACTGG------TTGGAATGAATTCATAACATCAAAGAAATTG 1252
           ** ******   ** ** *  **       ****  * ***   **** * ** *  ***

ARF13      ATTGTTGGGGATATATTTGTACTCCTTAGgtatgtttttgtcttttacat------ctc 1203
ARF14      GTTGAAGGAGATGTTATTGTATTCGTTAGgtataatattggttcttctaatatatctttg 1301
```

```
ARF22           GTTGCAGGGGATGTTATTGTATTCGTTAGgtatattattggttttttttatatagggatc 1315
ARF12           GTTGCAGGGGATGTCATCGTATTCGTTAGgtatattattgggtttttttatatatggatt 1216
ARF20           GTTAAAGGAGATGTTATTGTATTCGTTAGgtataatattgattcttgttatatatctt-- 1275
ARF15           GTTAAAGGAGATGTTATTGTATTCGTTAGgtataatattggttcttgttatatatttt-- 1258
ARF21           GTTAAAGGAGATGTTATTGTATTCGTTAGgtataatattggttcttgttatatatctt-- 1310
                 **   ** *** *  * *** ** ********  * ***  * **   **

ARF13           ttctcttttttttggatattagaatttaaactattttcttgttttttcagAGGAGAGAATG 1263
ARF14           ----tgttcttgagt-tttggactttaaactatatacttgtggttttagGGGAGAGACTG 1356
ARF22           ttttggttcttgagtttt-ggattttaaactatttacttgtggtttcagGGGAGAGACTG 1374
ARF12           ttttggttcttgagt-tttggactttaaactatttacttgtggtttcagGGGAGAGACTG 1275
ARF20           --ttggttttggagttttggacttttaaactatttacttgtggttttagGGGAGAGACTG 1333
ARF15           --ttggttcttgagttttggact-ttaaactatttacttgtggttttagGGGAGAGACTG 1315
ARF21           --ttgattcttgagttttggact-ttaaactatttacttgtgattttagGGGAGAGACTG 1367
                      ** *   *   *      ********* * *****  *** ** ******* **

ARF13           GGGAGTTACGATTTGGTATTAGGCGAGCAAAGCATCAACAAGGCCACATACCTTCATCAG 1323
ARF14           GAGAGTTACGAGTTGGTATCAGACGAGCAGGACATCAACAAGGGAATATACCTTCATCAA 1416
ARF22           GAGAGTTACGAGTTGGTATCAGACGAGCAGGACATCAACAAGGGAATATACCTTCATCAA 1434
ARF12           GAGAGTTACGAGTTGGTATCAGACGAGCAAGACATCAACAAGGGAATATACCTTCATCAA 1335
ARF20           GAGAGTTACGAGTTGGTATCAGACGAGCAAGACATCAACAAGGGAATATACCTTCATCAA 1393
ARF15           GAGAGTTACGAGTTGGTATCAGACGAGCAAGACATCAACAAGGGAATATACCTTCATCAA 1375
ARF21           GAGAGTTACGAGTTGGTATCAGACGAGCAAGACATCAACAAGGGAATATACCTTCATCAA 1427
                * ********* ******* ** ******   *********** * ************

ARF13           TAATATCAGCAAATTGTATGCAACATGGAGTAATAGCTTCAGTAGTGAATGCTTTTAAAA 1383
ARF14           TAGTATCAATAGAAAGTATGAGACATGGGATAATTGCTTCTGCAAAGCATGCTTTTGATA 1476
ARF22           TAATATCAATAGAAAGTATGAGACATGGGGTGATTGCTTCCGCAAAGCATGCTTTTGATA 1494
ARF12           TAGTATCAATAGATTGTATGAGACATGGGGTAGTTGCTTCCGCAAAGCATGCTTTTGATA 1395
ARF20           TAGTATCAATAGATTGTATGAGACATGGGGTAATTGCTTCCGCAAAGCATGCTCTTGATA 1453
ARF15           TAGTATCAATAGATTGTATGAGACATGGGGTAATTGCTTCCGCAAAGCATGCTTTTGATA 1435
ARF21           TAGTATCAATAGATTGTATGAGACATGGGGTAATTGCTTCCGCAAAGCATGCTTTTGATA 1487
                ** *****  * *  *****  ******  *  * ***** * *  * ***** ** * *

ARF13           CCAAATGCATGTTCAATGTGGTTTATAAGCCAAGgtgattcagaatagtacttaat--tt 1441
ARF14           ACCAATGTATGTTCATTGTGGTTTACAAGCCAAGgtaatttcaaagaaccatttgaactc 1536
ARF22           ACCAATGTATGTTCATTGTGGTTTACAAGCCAAGgtaatt-caaagaaccatttgaactc 1553
ARF12           ACCAATGTATGTTCACTGTGGTTTACAAGCCAAGgtaatt-caaagaaccatttgaactc 1454
ARF20           ACCAATGTATATTCATTGTGGTTTACAAGCCAAGgtaatt-caaagaaccatttgaactc 1512
ARF15           ACCAATGTATGTTCATTGTGGTTTACAAGCCAAGgtaatt-caaagaaccatttgaactc 1494
ARF21           ACCAATGTATATTCATTGTGGTTTACAAGCCAAGgtaatt-gaaagaaccatttgaactc 1546
                 * **** ** **** ********* ********** ***   ** *    **     *

ARF13           acattttacagtctgccaatatatcatgatattagcttcttcaaccaaatgtatgtttac 1501
ARF14           acattttagattatattgatttatcaataaattagtttcttcaacatgtaggtttatat- 1595
ARF22           acttttaagattatattgatttatcaatagattagttccttcaacatgtaggtttatata 1613
ARF12           acattttagattatattgatttatcaatagattagttccttcaacatgtaggtttatat- 1513
ARF20           acattttagattatattgatttatcaatagattagttccttcaacatataggtttatat- 1571
ARF15           acattttagattatattgatttatcaatagattagttccttcaacatataggtttatat- 1553
ARF21           acattttagattatattgatatatcaatagattagtcccttcaacatataggtttatat- 1605
                ** *** * * * *    ** *****  * *****   *******     *  *  *

ARF13           atttgtttttttcatataatattagttcaagtcaatttgttataagctatgacaaatttg 1561
ARF14           ---tttgtgtctttataagtatcagGTCAAGTCAATTTATTGTCAGTTACGACAAATTCT 1652
ARF22           tttttgtgtctttataagtatcagATCAAGTCAATTTATTGTCAGTTACGACAAATTCT 1673
ARF12           ---tttgtgtctttataagtatcagGTCAAGTAAATTTATTGTCAGTTACGACAAATTCT 1570
ARF20           ---tttgtgtctttctaagtatcagGTCAAGTCAATTTATTGTCAGTTACGACAAATTCT 1628
ARF15           ---tttgtgtttttataagtatcagGTCAAGTCAATTTATTGTCAGTTACGACAAATTCT 1610
ARF21           ---tttgtgtctttataagtatcagGTCAAGTCAATTTATTGTCAGTTATGACAAATTCT 1662
                   **  * * *     * *** ** ****** ***** ** * ** ** ********

ARF13           ttgatgcaatgaacaataattacattgttggttcgagatttagGATGCAGTTTGAGGGTA 1621
ARF14           TAGACGTTGTGAACAATAAGTTCAATGTCGGTTCAAGATTTACAATGCGGTTTGAGGGTG 1712
ARF22           TAGATGCTGTGAACAATAAGTTCAATGTTGGTTCAAGATTTACAATGCGGTTTGAGGGTG 1733
ARF12           TGGACGCTGTGAACAATAAGTTCAATGTCGGTTCAAGATTTACAATGCGGTTAGAGGGTG 1630
ARF20           TAGACGCTATGAACAATAAGTTCATTGTCGGTTCAAGATTTACAATGCGGTTTGAGGGTG 1688
ARF15           TAGACGCTGTGAACAATAAGTTCAATGTCGGTTCAAGATTTACAATGCGGTTTGAGGGTG 1670
ARF21           TAGACGCTGTGAACAATAAGTTTAATGTCGGTTCAAGATTTACAATGCGGTTTGAGGGTG 1722
```

```
           * ** *   ********** *  * *** ***** *******  **** *** ******

ARF13      AGGATTTTTCTGAAAAAAGgtaaaaaatttaatcaaaactttttttctct---------a 1672
ARF14      ATGATTTCTCTGAAAGAAGgtaacattagatgaaaaaaaaa-attgcttaaatatttgg 1771
ARF22      ATGATTTCTCTGAAAGAAGgtaacattagatgaaaactttttctacttaaatatatttg 1793
ARF12      ATGATTTCTCTGAAAGAAGgtaacattagatgaaatttttttctgcttaaatatattta 1690
ARF20      ATGATTTCTCTGAAAGAAGgtaacattagatgaaaaaaaatttctgcataaatatattta 1748
ARF15      ATGATTTATCTGAAAGAAGgtaacattagatgaaaaagaatttctgcataaatatattta 1730
ARF21      ATGATTTCTCTGAAAGAAGgtaacattagatgaaatttattttctgcataaatatattta 1782
           * ***** ******* ******* *         *         * *

ARF13      tttccaaaatttgttatttgcataacacattttgttttatttactaagATACGATGGGAC 1732
ARF14      taatgtctaatattatctgtgataacacattttgttctatcgattaagATCTTTTGGGAC 1831
ARF22      taatgtttaatattatctatgataacacattttgttctattgattaagATATTTTGGGAC 1853
ARF12      taatgtctaatattatctatgataacacatattgttcaattgattaagATGTTTTGGGAC 1750
ARF20      taatgtttaatattatcaatgataacacattttgttcaattgattaagATATTTTGGGAC 1808
ARF15      taatgtctaatattatctatgataacacattttgttcaattgattaagATATTTTGGGAC 1790
ARF21      taatgtctaatattatttatgataacacattttgttcaattgattaagATATTTTGGGAC 1842
           *       * *  *       ********* *****  **  * ******    ******

ARF13      GATTATTGGTGTAAATGACATGTCTCCTCATTGGAAGGATTCAGAATGGCGAAGCCTAAA 1792
ARF14      AATTATTGGAGTTAGTGATTTCTCTCCTCATTGGAAGTGTTCAGAGTGGCGCAGCTTAGA 1891
ARF22      AATTATTGGAGTTAGTGATTTCTCTCCTCATTGGAAGTGTTCAGAGTGGCGCAACTTAGA 1913
ARF12      AATTATTGGAGTTAGTGATTTCTCTCCTCATTGGAAATGTTCAGAGTGGCGCAGCTTAGA 1810
ARF20      AATTATTGGAGTTAATGATTTCTCTCCTCATTGGAAGTGTTCAGAGTGGCGCAGCTTAGA 1868
ARF15      AATTATTGGAGTTAGTAATTTCTCTCCTCATTGGAAGTGTTCAGACTGGCGCAGCTTAGA 1850
ARF21      AATTATTGGAGTTAGTGATTTCTCTCCTCATTGGAAGTGTTCAGAGTGGCGCAGCTTAGA 1902
            ******** **  * * *  * **************   ****** ***** * * ** *

ARF13      Agtaatataatcttcatactttcatgac-atctttcaaaatacatttttgagtgtaataa 1851
ARF14      AGTAAAATTTCATTCACACTTTATAACATTATTTTAGAAATACGTTTTTGAGTGCAATAA 1951
ARF22      Agtaaaatttcattcacactttaaaacatcattttaggaatacgtttttgaatgcaataa 1973
ARF12      Agtaaaatttcattcacactttataacattattttaggaatacgtttttgagtgcaataa 1870
ARF20      Agtaaaatttcattcacactttataacattcttttaggaatacgtttttgagtgcaataa 1928
ARF15      Agtaaattttcattcacactttataacattcttttaggaatacgtttttgagtgcaataa 1910
ARF21      Agtaaaatttcattcacactttataacattcttttaggaatacgtttttgagtgcaataa 1962
           *****  *    **** *****          ***   ***** ******* ** *****

ARF13      ataacatatgactaatcaactatgtttttgtagGTGCAATGGGACGAGCTTTCACCATTT 1911
ARF14      ATAGCATATCACTAATTAACTCTAATGTTGCAGGTGCAGTGGGATGAATTTGCATCATTT 2011
ARF22      -------------------atttaatcttgcagGTGCAGTGGGATGAATTTGCATCATTT 2014
ARF12      ataacatatcactaattaactttaattttgcagGTGCAGTGGGATGAATTTACATCATTT 1930
ARF20      ataacatatcactaattaactttaattttgcagGTTCAGTGGGATGAATTTGCATCATTT 1988
ARF15      ataacatatcactaattaactttaattttgcagGTGCAGTGGGATGAATTTGCATCATTT 1970
ARF21      ataacatatcactaattaactttaattttgcagGTGCAGTGGGATGAATTTGCATCATTT 2022
                               * *  * *** **** ** ***** **  ** ** *****

ARF13      CTAAGACCTAATCAGGTTTCACCTTGGGACATCGAGCATCTAATTCCTTCGTCAGATATC 1971
ARF14      CCGAGACCAAACCAAGTTTCACCATGGGACATCGAACATTTAACGCCTTGGTCAAATGTT 2071
ARF22      TCGAGACCTAATAAAGTGTCACCCTGGGAGATCGAACATTTAATGCCTGCGTTAAATGTT 2074
ARF12      CCGGGACCTAAAAAAGTGTCACCCTGGGACATCGAACATTTAATGCCTGCAATAAATGTT 1990
ARF20      TCGAGACCTAATAAAGTGTCACCCTGGGAGATCGAACATTTAATGTCTGCGTTAAATGTT 2048
ARF15      TTGAGACCTAATAAAGTGTCACCCTGGGAGATCGAACATTTAATGCCTGCGTTAAATGTT 2030
ARF21      TCGAGACCTAATAAAGTGTCACCCTGGGAGATCGAACATTTAGTGCCTGCGTTAAATGTT 2082
               **** **  * ** ***** ***** ***** *** **    **     * ** *

ARF13      TCTCAATCAAGTTTGAAAAAGAAAAAACATTGGCTTCAATTGAATGAAATTGgtaaaagg 2031
ARF14      TCCAGGTCATCTTTTCTCAAGAACAAGCGTTCACGAGAAGTTAATGAAATCGGTATAAAA 2131
ARF22      CCGCGACCATCTTTGCTCAAGAACAAGCGTTTACGAGAAGTTAATGAAATCGgtataaaa 2134
ARF12      CCGCGATCATTTTTGCTCAAGAACAAGCGTTTACGAGAAGTTAATGAAATCGgtataaaa 2050
ARF20      CCGCGATCATCTTTGCTCAAGAACAAGCGTTTACGAGAAGTTAATGAATTCGgtataaaa 2108
ARF15      CCGCGATCATCTTTTCTCAAGAACAAGCGTTTACGAGAAGTTAATGAATTCGgtataaaa 2090
ARF21      CCGCGATCATCTTTGCTCAAGAACAAGCGTTTACGAGAAGTTAATGAATTCGgtataaaa 2142
            *     **  ***    ***** ** * **  *   ** * ****** * **** **

ARF13      aaacatgcttataatcattggtttgataagtttatgcttcctcatgaaaatatctttctc 2091
ARF14      AAAGACACTTATAATCTTC-------------------TATTTAATGAATTTAAGATTC 2171
ARF22      agagactcttataatcttttatttaaagaattaatcttctatttaatgaatttgagattc 2194
```

```
ARF12      aaagactcttataatcttttatttaaagaattaatcttctatttaatgaatttggtattc 2110
ARF20      atacactcttataatcttttatttaaaggattaatcttctatttaatgaatttgagactc 2168
ARF15      atacactcttataatcttttatttaaagaattaatcttctatttaatgaatttgagattc 2150
ARF21      agagactcttataatcttttatttaaagaattaatcttctatttaatgaatttgagattc 2202
           * * *  ********* *                       *      *** *     **

ARF13      attctagatat-------------tttcatatatagGTGCAACATTATCGAATCTTTGG- 2137
ARF14      TTTAATACCTTCTCAATATACCTATTTTATATACAGGTTCTTCCTCATCACATCTCTTGC 2231
ARF22      ttaaataccttctcaatattcctgttttatatacagGTTCTTCATCATCACATCTCTTGC 2254
ARF12      ttgaataccttctcaataatcctattttatatacagGTTCTTCATCATCACATCTCTTAC 2170
ARF20      ttgaataccttctcaatattcttgttttatatacatgttcttcatcatcacatatcttgc 2228
ARF15      ttgaataccttctcaatattcttgttttatataaagGTTCTTCATCATCACATCTCTTGC 2210
ARF21      ttaaattccttctcaatattcatgttttatatacagGTTCTTCATCATCACATCTCTTGC 2262
            *        *             *** ***** * ** *  * * ***  ** * *

ARF13      --------------ACATGCCAAGAAATTGGACAACGGAGCATGAATTCTCCTATAAGTG 2183
ARF14      CTCCTACATTGACACAAGGACAAGAAATTGGACAACAAAGCATGGCCACCCCGATGAATA 2291
ARF22      CTCCTATATTGACACAAGGACAAGAAATTGGCCAACTAAGCGTGGCCTCCCCGATGAATA 2314
ARF12      CTCCTATATTGACACAAGGACAAGAAAATGAACAACTAAGTGTTGCCTCTCCGATGAATA 2230
ARF20      ctcctatattgacacaagGACAAGAAATTGGACAACTAAGCGTCGCCTCCCCGATGAATA 2288
ARF15      CTCCTATATTGACACAAGGACAAGAAATTGGACAACTAAGCGTGGCCTCCCCGATGAATA 2270
ARF21      CTCCTATATTGACACAAGGACAAGAAATTGGACAACTAAGCGTGGCCTCCCCGATGAATA 2322
                           * * ******* **  ****  **  *     * ** ** * *

ARF13      TTCCTGAGTTTAGTTATCCCAATGCAATTGAAGATTCAAAGTTTCTTTCTGGTTTGCTAC 2243
ARF14      TTT---CTCTTCGTTATCGTGATATTACTGAAGATGCTATGACTCCTTCTAGATTGCTAA 2348
ARF22      TTT---CTCTCACTTATCGTGATACAACTGAAGATGTTATGAATCCTTCTAGATTGCTAA 2371
ARF12      TTT---CTCTTCGTTATCGTGATGCAACTGAAGATGCTATGAATCCTTCTAAATTGCTAA 2287
ARF20      CTT---CTCTTCGTTATCGTGATACAACTGAAGATGCTATGAATCCTTCTAGATTGCTAA 2345
ARF15      TTT---CTCTTCTTTATCGTGAAACAACAGAAGATGCTATGAATCCTTCTAGATTGCTAA 2327
ARF21      TTT---CTCTTCGTTATCGTGATACAACTGAAGCTGCTATGAATCCTTCTAGATTGCTAA 2379
            *       *   *****   *    *  **** *   * *  ** ****   ******

ARF13      TGAATCACTCACTCCTAGCCATACCTAATGAAAACTATAACAGCGACCAAATGATTCAAC 2303
ARF14      TGAGCTACCCTGTCCAACCAATGGCCAAACTAAAT---TACAATAAT---GTGGTTACAC 2402
ARF22      TGAGCTACCCTGTCCAACCAATGCCCAAACTAAAT---TACAATAACCAAATGGTTACAC 2428
ARF12      TGAGCTACCCTGTCCAACCAATGCCCAAACTAAAT---TACAATAACCAAATGGTTACAG 2344
ARF20      TGAGCTACCCTGTCCAACCAATGCCCAAACTAAAT---TACAATAACCAAATGGTTACAC 2402
ARF15      TGAGCTACCCTGTCCAACCAATGCCCAAACGAAAT---TACAATAACCAAATGGTTACAC 2384
ARF21      TGAGCTACCCTGTCCAACCAATGCCCAAACTAAAT---TACAATAACCAAATGGTTACAC 2436
           ***   ** *  *** * * **  * **   ***     ***   *     ** **  *

ARF13      CAAGGAAAGAAGATATAACAACTGAAGCAACCACTAGTTGCCTCTTGTTTGGAGTTGATC 2363
ARF14      CAATAGAAGAAAATATAACAACCAATGCAGTCGCTAGTTTAGGTTGTTTGGAGTCTCTC 2462
ARF22      AAATAGAAGAAAATATAACAACCAAGACTGGCACTAATTTTAGGCTGTTTGGAGTCTCAC 2488
ARF12      AAATGGAAGAAAATATAACAACCAAGACAGGCACTAATTTTAGGCTGTTTGGAGTCACTC 2404
ARF20      AAATAGAAGAAAATATAACAACCAAGGCAGTCACTAATTTTAGGCTGTTTGGAGTCTCTC 2462
ARF15      AAATAGAAGAAAATATAACAACCAAGGCAGGCACTAATTTTAGGTTGTTTGGAGTCTCTC 2444
ARF21      AAATAGAAGAAAATATAACAACCAAGGCAGGCACTAATTTTAGGCTGTTTGGAGTCACTC 2496
            **   ***** **********  *  *   * *** **      **********    *

ARF13      TGACCAAAgtatcaaagagcaaagattccatctgtccaattgaatcatgcaaaaaatcag 2423
ARF14      TGGCCACTCCTTCGGTGATCAAAGATCCCGTTGAACAAATTGG----------------- 2505
ARF22      TGGTCACTCCTTCAGTGATCAAAGATCCCATTGAAGAAATTGG----------------- 2531
ARF12      TGGACACTCCTCCGGTGATCAAAGATCCCATTGAAGAAATTGG----------------- 2447
ARF20      TAGCCATTCCTCTAGTGATCAAAGATCCCATTGAAGAAATTGG----------------- 2505
ARF15      TGGCCACTCCTCCGGTGATCAAAGATCCCATTGAACAAATTGG----------------- 2487
ARF21      TGGACACTCCTCCGATGATCAAAGATCCCATTAAACAAATTGG----------------- 2539
           *   **          ** ******* ** *      *****

ARF13      aaatttcaaaactcaaaaatcaaaaagcaaccactagttgcctcaagataaaaagtttga 2483
ARF14      ---------------------CTTGGAGATTTCTAGACTTACTCAGGAAAAAAAGTTTGG 2544
ARF22      ---------------------CTCGGAGATTTCGAAACTTACTGAAGGAAAAAAGTTTGG 2570
ARF12      ---------------------CTCGGAGATTTCGAAACTTACTGAAGGAAAAAAGTTTGG 2486
ARF20      ---------------------CTCGGATATTTCGAAACTTACTGAAGGAAAAAAGTTTGG 2544
ARF15      ---------------------CTCGGATATTTCGAAACTTACTGAAGGAAAAAAGTTTGG 2526
ARF21      ---------------------CTCGGATATTTCGAAACTTACTGAAAGAAAAAAGTTTGG 2578
                                       *         *  ** *    **********
```

```
ARF13      ccaaacccaacctctgagatcaccaaaagaggtccaaagcacggaattcaattttactag 2543
ARF14      TCAAAGCCAAATTTTGAGATCACCAACAGAGATCCAAAGCAAGCAGTTCAGTTCTACCAG 2604
ARF22      TCAAAGCCAAACTTTGAGATCACCAACAGAGATCCAAAGCAAGCAGTTCAGTTCTACTAG 2630
ARF12      TCTAAGCCAAACTTTAAGATCACCAACAGAGATCCAAAACAAGCAGTTCAGTTCTAGTAG 2546
ARF20      TCAAAGCCAAACTTTGAGATCACCAATAGAGATCCAAAGCAAGCAGTTTGGTTCTACTAG 2604
ARF15      TCAAAGCCAAACTTTGAGATCACCAACAAAAATCCAAAGCAAGCAGTTCAGTTCTACTAG 2586
ARF21      TCAAAGCCAAACTTTGAGATCACCAATAGAGATCCAAAGCAAGCAGTTCAGTTCTTCTAG 2638
            * ** ****  * * ********** * *  ****** ** * * **   ** *   **

ARF13      aagtcgtattaaagtaagcataaaatcattatatctgtaacatatgacttttttttaaa 2603
ARF14      AACTTGTACCAAAgtaagtataagttcaatat---------------------------- 2636
ARF22      AACTTGTACCAAAgtaagtataagatcaatat---------------------------- 2662
ARF12      AACTTGTACCAAAgtaagtataagatcaatat---------------------------- 2578
ARF20      AACTTGTACCAAAgtaagtataagatcaat------------------------------ 2634
ARF15      AACTTGTACGAAAgtaagtataagatcaatat---------------------------- 2618
ARF21      GACTTGTACCAAAgtaagtataagatcaatat---------------------------- 2670
            * * ***  ******** ****  *** *

ARF13      atgtgaaactagtgcattcaagcaagcattcgccgaaaaaaatatattttaagaaatttt 2663
ARF14      ------------------------------------------------------------ 2636
ARF22      ------------------------------------------------------------ 2662
ARF12      ------------------------------------------------------------ 2578
ARF20      ------------------------------------------------------------ 2634
ARF15      ------------------------------------------------------------ 2618
ARF21      ------------------------------------------------------------ 2670

ARF13      gataagaaacatacatattaggtaacttagtatttttagatatcaaatatatatttgata 2723
ARF14      -------------------------att---cttatt---------------ctttatat 2653
ARF22      -------------------------att---cttatt---------------ctttatat 2679
ARF12      -------------------------act---cttatt---------------atttatat 2595
ARF20      -------------------------att---cttatt---------------ccttatat 2651
ARF15      -------------------------att---cttatt---------------ccttatat 2635
ARF21      -------------------------att---cttatt---------------ccttatat 2687
                                      *    ** **                  **

ARF13      agaaaatttcgtaaagctttcacatgattttgatgtggtatgttctaacagGTTCATATG 2783
ARF14      atgaaatttttctcattatactcaactcacgcaatcttaatatggaatagGTTCAAATG 2713
ARF22      atgaaactttttctcattgtactcaactcacgcaatcttgatatggaacagGTTCAAATG 2739
ARF12      atataactttttctcattgtactcaactcacgcaatcttgatatggaacagGTTCAAATG 2655
ARF20      atgaaactttttctcattgtactcaactcatgcaatcttgatatggaacagGTTCAAATG 2711
ARF15      atgaaactttttctcattatactcacctcacacaattttgatatggaacagGTTCAAATG 2695
ARF21      atgaaactttttctcattgtactcaactcacacaatctttatatggaacagGTTCAAATG 2747
           *   ** **  *     * *       *       *  *    *  ** ******* ***

ARF13      CAAGGTGTAGCCATAAGTAGAGCTGTGGATTTAACTGCTATGCATGGATACAATCAGCTG 2843
ARF14      CAAGGTGTAACAATAGGAAGAGCTGTGGATTTAAGTGTTCTTAATGGATATGATCAGTTA 2773
ARF22      CAAGGTGTAACAATAGAAAGAGCTGTGGATTTAAGTGTTCTGAATGGATATGATCAGCTA 2799
ARF12      CAAGGTGTAACAATAGGAAGAGCTGTGGATTTAAGTGTTCTTAATGGATATGATCAGCTA 2715
ARF20      CAAGGTGTAACAATAGGAAGAGCTGTGGATTTAAGTGTTCTTAATGGATATGATCAGCTA 2771
ARF15      CAAGGTGTAACAATAGGAAGAGCTGTGGATTTAAGTGTTCTTAATGGATATGATCAGCTA 2755
ARF21      CAAGGTGTAACAATAGGAAGAGCTGTGGATTTAAGTGTTCTTAATGGATATGATCAGCTA 2807
           ********* * ***   **************** ** * *  *******  ***** *

ARF13      ATACAAAAACTGGAAGAACTCTTTGATCTCAAAGACGAGTTACGAACTCGCAATCAATGG 2903
ARF14      ATACTAGAATTGGAGAAGCTCTTCGATCTCAAAGGCCAATTGCAAGCTCGCAACCAATGG 2833
ARF22      ATACTAGAATTGGAGGAGCTCTTCGATCTCAAAGGCCAATTACAAACTCGCAACCAATGG 2859
ARF12      ATACTAGAATTGGAGAAGCTCTTTGATATCAAAGGTCAATTGCAAACTCGCAACCAATGG 2775
ARF20      ATACTAGAATTGGAGAAGCTCTTTGATCTCAAAGGCCAACTACAAACTCGCAACCAATGG 2831
ARF15      ATACTAGAATTGGAGAAGCTCTTCGATCTCAAAGGCCAATTACAAACTCGCAACCAATGG 2815
ARF21      ATACTAGAATTGGAGAAGCTCTTTGATATCAAAGGCCAATTACAAACTCGCAACCAATGG 2867
           **** * ** ****  * ***** *** ******   *  * * * ******* ******

ARF13      GAAATAGTTTTTACAAACAATGAAGGAGCTGAGATGCTTGTCGGGGATGATCCATGGCCg 2963
ARF14      GAAATAGCTTTCACAAATAATGAAGAGGATAAGATGCTTGTTGGAGAAGATCCATGGCCg 2893
ARF22      GAAATAGCTTTCACAGATAGTGACGATGATAAGATGCTTGTTGGAGACGATCCATGGCCg 2919
ARF12      GAAATAGCTTTCACAGATAGTGACGAAGATAAGATGCTTGTTGGAGACGATCCATGGCCg 2835
```

```
ARF20      AAAATAGCTTTCACAGATAGTGACGGGTATGAGATGCTTGTTGGAGACGATCCATGGCCg 2891
ARF15      AAAATAATTTTCACAGGTAGTGACGAGGATGAGATGCTTGTTGGAGACGATCCATGGCCg 2875
ARF21      AAAATAGCTTTCACAGATAGTGACGGGTATGAGATGCTTGTTGGAGACGATCCATGGCCg 2927
            *****  *** ***   * *** *    * ********** ** ** ************

ARF13      tatgttttctttctctttctatatattgaaacccatgttaaaatattgagaaaatgtcat 3023
ARF14      taagttttctttctatttctatcgattgaaagattagttaaaatcttgagagacattgat 2953
ARF22      taagtttcttttctatttctatcgattgaaagattagttaaaatcttgagagacgttgat 2979
ARF12      taagtttcctttctatttctatcgattgaaagattagttaacatcttgagagacattgat 2895
ARF20      tgagtttcctttctatttctattgattaaaagattagttaaaatcttgagagacattgat 2951
ARF15      tgagtttcctttctatttctatcgattgaaagattagttaaaatcttgagaggcattgat 2935
ARF21      tgagtttccttttatttctattgattgaaagattagttaaaatcttgagagacattgat 2987
           *  ****  *** * *******  *** ***     ***** ** ******     * **

ARF13      tttgaaa-------attgtgtgcatccagTGAGTTCTGCAATATGGCGAAAAGAATATTC 3076
ARF14      atttcatttttt---ttttttcatgtagTGAATTCTGCAACATGGTGAAGAAGATATTC 3010
ARF22      atttcatttttttgtgttgtttgcatatagTGAATTCTGCAACATGGTGAAGAAGATATTG 3039
ARF12      attttattttttgtgttgtttgcatgtagTGAATTCTGCAACATGGTGAAGAAGATATTC 2955
ARF20      atttcatt-tttgtgttgtttgcatgtagTGAATTCTGCAAAATGGTGAAGAAGATATTG 3010
ARF15      atttcatt-tttgtgttgtttgcatgtagTGAATTCTGCAACATGGTGAAGAGGATATAT 2994
ARF21      atttcatt-tttgtgttgtttgcatggagTGAATTCTGCAAAATGGTGAAGAAGATATTG 3046
            **  *         ** * * ***  ***** ******** **** *** *  ****

ARF13      ATATGCTCAAAAGAGGAGATAAAGAAAATGAAGTTGAAGAACAAATTCTTTCAACCTGAA 3136
ARF14      ATATATTCAAAAGAGGAGGTCAAAAACTTGAAGTCCAGGAAGAGTCTCTCAAGTTGA--- 3067
ARF22      ATATTCAAAAGAGGA--GGTCAAAAACTTGAAGTCCAGTAA------------------- 3078
ARF12      ATTCAAAAGAGGAGGTAA------------------------------------------ 2973
ARF20      ATATATTCAAAAGAGGAGGTCAAAAACCTAAAGTCCAGTAAGAGTCTCTCAAGTTGA--- 3067
ARF15      ATTCAAAAGAGGAGGTAA------------------------------------------ 3012
ARF21      ATATATTCAAAAGAGGAGGTCAAAAACCTGAAGTCCAGTAAGAGTCTCTCAAGTTGA--- 3103
           **       *

ARF13      TCAAAAGCTTTAACATCTTCAGACGTACCACCAAACGTCACAGATAACTAA    3187
ARF14      ---------------------------------------------------    3067
ARF22      ---------------------------------------------------    3078
ARF12      ---------------------------------------------------    2973
ARF20      ---------------------------------------------------    3067
ARF15      ---------------------------------------------------    3012
ARF21      ---------------------------------------------------    3103
```

Legend

Start and stop codon

B3 domain

Auxin response factor domain

Aux/IAA domains

Exons are in upper case / Introns are in lower case
